# Balancing Carrier Dynamics in Oxygen‐Vacancy‐Tuned Amorphous Ga_2_O_3_ Thin‐Film Self‐Powered Photoelectrochemical‐Type Solar‐Blind Photodetector Arrays for Underwater Imaging

**DOI:** 10.1002/advs.202407822

**Published:** 2024-09-30

**Authors:** Ke Ding, Hong Zhang, Jili Jiang, Jiangshuai Luo, Rouling Wu, Lijuan Ye, Yan Tang, Di Pang, Honglin Li, Wanjun Li

**Affiliations:** ^1^ Chongqing Key Laboratory of Photo‐Electric Functional Materials and Laser Technology College of Physics and Electronic Engineering Chongqing Normal University Chongqing 401331 P. R. China

**Keywords:** amorphous Ga_2_O_3_, oxygen vacancies, photoelectrochemical‐type photodetectors, self‐powered, solar‐blind underwater imaging

## Abstract

Underwater imaging technology plays a pivotal role in marine exploration and reconnaissance, necessitating photodetectors (PDs) with high responsivity, fast response speed, and low preparation costs. This study presents the synergistic optimization of responsivity and response speed in self‐powered photoelectrochemical (PEC)‐type photodetector arrays based on oxygen‐vacancy‐tuned amorphous gallium oxide (a‐Ga_2_O_3_) thin films, specifically designed for solar‐blind underwater detection. Utilizing a low‐cost one‐step sputtering process with controlled oxygen flow, a‐Ga_2_O_3_ thin films with varying oxygen vacancy (V_O_) concentrations are fabricated. By balancing the trade‐offs among electrocatalytic reactions, charge transfer, carrier recombination, and trapping, both the responsivity and response speed of a‐Ga_2_O_3_‐based self‐powered PEC‐PDs are simultaneously improved. Consequently, the optimized PEC‐PDs demonstrated exceptional performance, achieving a responsivity of 33.75 mA W^−1^ and response times of 12.8 ms (rise) and 31.3 ms (decay), outperforming the vast majority of similar devices. Furthermore, a pronounced positive correlation between anomalous transient photocurrent spikes and the concentration of V_O_ defects is observed, offering compelling evidence for V_O_‐mediated indirect recombination. Finally, the proof‐of‐concept solar‐blind underwater imaging system, utilizing an array of self‐powered PEC‐PDs, demonstrated clear imaging capabilities in seawater. This work provides valuable insight into the potential for developing cost‐effective, high‐performance a‐Ga_2_O_3_ thin‐film‐based PEC‐PDs for advanced underwater imaging technology.

## Graphical abstract

Simultaneously enhancing responsivity and response speed in oxygen‐vacancy‐tuned amorphous Ga_2_O_3_ thin‐film photoelectrochemical‐type self‐powered photodetectors involves balancing trade‐offs among electrocatalytic reactions, charge transfer, carrier recombination, and trapping. Transient photocurrent is closely associated with V_O_ defects. The proof‐of‐concept underwater imaging system utilizes self‐powered photodetectors to demonstrate clear solar‐blind imaging in seawater, with potential future applications in marine exploration and reconnaissance.

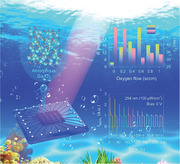

## 1 Introduction

Underwater imaging technology is widely used in target reconnaissance, detection, and identification. Most underwater photodetectors (PDs) predominantly respond to visible and UV light.^[^ ^1^, ^2^ ^]^ However, background radiation from the sun or other light sources can cause significant interference, increasing the burden on signal processing systems and potentially leading to false alarms.^[^ ^3^, ^4^ ^]^ Short‐wave ultraviolet (UV‐C) light in the 200–280 nm range is absorbed by the ozone layer and stratosphere, preventing it from reaching the Earth's surface and creating a solar‐blind zone.^[^ ^5^ ^]^ This natural absorption provides inherent shielding for photodetection systems operating within this wavelength range.^[^ ^6^, ^7^, ^8^ ^]^ As a result, solar‐blind UV‐C underwater imaging systems can operate continuously, benefiting from extremely low background light noise and significantly reducing the complexity of signal processing. New photoelectrochemical‐based photodetectors (PEC‐PDs) are naturally suited for underwater use and can be directly exposed to the electrolyte without needing additional corrosion‐resistant encapsulation.^[^ ^9^, ^10^, ^11^ ^]^ They operate primarily at the semiconductor–electrolyte (S–E) interface, where the built‐in electric field facilitates the direct separation of photogenerated carriers, allowing the devices to function without an external bias voltage. Additionally, with electrodes buried beneath the photosensitive layer, there is no need for expensive surface precious metal electrode deposition or complex photolithography processes. This significantly reduces preparation costs and prevents light obstruction by the electrodes. Thus, developing high‐performance, self‐powered solar‐blind UV‐C PEC‐PDs is crucial for advancing the next generation of underwater imaging technology.

Recently, ultra‐wide bandgap semiconductors like AlGaN^[^ ^12^, ^13^, ^14^, ^15^, ^16^, ^17^ ^]^ and Ga_2_O_3_^[^ ^18^, ^19^, ^20^ ^]^ have been successfully used in self‐powered PEC‐PDs for solar‐blind UV‐C detection. Among these, Ga_2_O_3_ has gained significant attention due to its excellent chemical stability, natural solar‐blind photosensitivity range, and ability to avoid complex alloying processes.^[^ ^19^ ^]^ Various forms of Ga_2_O_3_ have been reported in PEC‐PDs, including α‐phase Ga_2_O_3_, β‐phase Ga_2_O_3_, and amorphous Ga_2_O_3_ (a‐Ga_2_O_3_). α‐Ga_2_O_3_ as a Photoanode: α‐Ga_2_O_3_ nanorod arrays (NRAs) are widely used as photoanodes. Researchers typically grow GaOOH nanoarrays on FTO substrates using low‐cost methods like hydrothermal or water bath techniques. These are then converted to α‐Ga_2_O_3_ NRAs through low‐temperature annealing.^[^ ^18^ ^]^ Additionally, heterojunctions with GaOOH,^[^ ^20^ ^]^ Cu_2_O,^[^ ^21^ ^]^ and Al_2_O_3_^[^ ^22^ ^]^ have been constructed to enhance performance. β‐Ga_2_O_3_ as a Photoanode: β‐Ga_2_O_3_ is highly stable and has garnered attention in PEC‐PDs, although high‐quality material preparation requires high temperatures.^[^ ^23^ ^]^ Researchers have successfully constructed β‐Ga_2_O_3_ NRAs PEC‐PDs using high‐temperature‐resistant substrates like Si^[^ ^24^ ^]^ and GaN,^[^ ^25^ ^]^ which generally outperform other devices. Recently, β‐Ga_2_O_3_ single‐crystal blocks have also been applied in PEC‐PDs research.^[^ ^26^ ^]^ Amorphous Ga_2_O_3_ as a Photoanode: Our group has developed a‐Ga_2_O_3_ photoanodes by coating amorphous Ga_2_O_3_ on 3D carbon fiber paper,^[^ ^27^ ^]^ conductive carbon cloth,^[^ ^28^ ^]^ and silver nanowires.^[^ ^29^ ^]^ These devices perform comparably to those based on other crystalline phases of Ga_2_O_3_ and offer the additional advantage of flexibility, demonstrating the excellent potential of amorphous Ga_2_O_3_‐based PEC‐PDs.

The excellent response performance of photodetectors stems from significant photodetection gain: G = τ/t_L_, where τ is the photogenerated carrier lifetime and t_L_ is the carrier transit time.^[^ ^30^ ^]^ Unfortunately, this mechanism often results in a longer photocarrier lifetime, leading to an extended response time.^[^ ^31^, ^32^ ^]^ Amorphous Ga_2_O_3_ has many defects, such as oxygen vacancies (V_O_), due to its disordered structure. In Ga_2_O_3_‐based photodetectors, V_O_ defects form carrier traps that prevent the recombination of holes and electrons, significantly prolonging the carrier lifetime and resulting in high photoconductivity gain.^[^ ^33^, ^34^, ^35^ ^]^ However, these V_O_ defects also cause persistent photoconductivity (PPC) after illumination ends, posing a challenge to achieve both high responsivity and fast response speed in solid‐state devices. Improved responsivity can also be achieved by reducing the carrier transit time, often through external bias voltages, but this is impractical in harsh environments where power support is unavailable.^[^ ^36^, ^37^ ^]^ Therefore, balancing responsivity and response speed without external bias voltage remains a critical scientific problem. Self‐powered PEC‐PDs not only involve the physical processes of solid‐state devices but also complex chemical reactions at the semiconductor–electrolyte (S–E) interface, leading to more intricate carrier dynamics. Exploring the effects of V_O_ defects on the responsivity and response speed of amorphous Ga_2_O_3_‐based PEC‐PDs is thus of great research significance to understand their intrinsic mechanisms and improve device performance.

In this work, we prepared a series of amorphous Ga_2_O_3_ thin‐film‐based self‐powered PEC‐PDs with an oxygen vacancy concentration gradient. This was achieved by controlling the oxygen flow parameter using a low‐cost RF magnetron sputtering technique. By effectively regulating the oxygen vacancies, we simultaneously optimized the responsivity and response speed of the self‐powered devices and elucidated the intrinsic mechanism by which oxygen vacancies affect performance. The self‐powered PEC‐PDs with optimal performance exhibited excellent photodetection and could operate stably and continuously in seawater. Additionally, we found that the controversial transient photocurrent is closely related to oxygen vacancies. Finally, we demonstrated a proof‐of‐concept for self‐powered solar‐blind UV‐C underwater imaging using an array of amorphous Ga_2_O_3_ thin‐film‐based PEC‐PDs. This work provides a reliable solution for developing self‐powered Ga_2_O_3_‐based PEC‐PDs with high responsivity and fast response speed, and it highlights the great potential of amorphous Ga_2_O_3_ thin films for self‐powered solar‐blind UV‐C underwater imaging.

## 2 Results and Discussions

### 2.1 Oxygen Vacancy Defects in Amorphous Ga_2_O_3_

**Figure 1:**
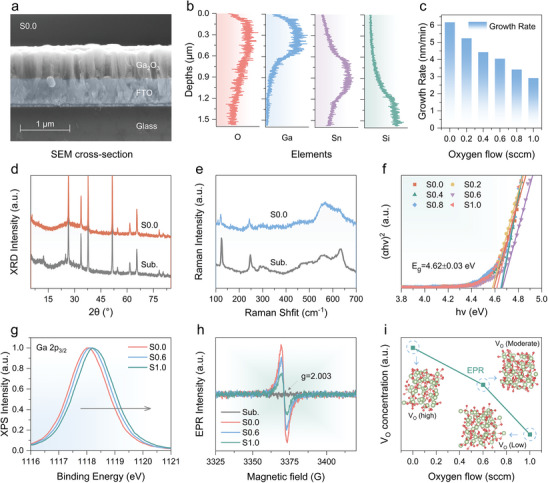
Material Characterizations. a) Cross sectional SEM image and b) Line‐scan EDS mapping of the Ga_2_O_3_ film. c) Dependence of film growth rate on oxygen flow. d) XRD patterns, e) Raman scattering spectra, and f) Plot of (αhν)^2^ versus hν for Ga_2_O_3_ films. g) XPS Ga 2p_3/2_ core‐level spectra and h) EPR spectra of S0.0, S0.6, and S1.0. i) V_O_ concentration as a function of oxygen flow. Models of amorphous Ga_2_O_3_ with different V_O_ concentrations can be found in Note S1 (Supporting Information).

The separation of photogenerated carriers in self‐powered photodetectors primarily relies on the built‐in electric field.^[^ ^19^ ^]^ In self‐powered PEC‐PDs, carriers at the photoanode are separated by the built‐in electric field at the solid‐liquid interface, with photogenerated electrons traveling to the back electrode via the n‐type photosensitive material.^[^ ^38^ ^]^ As a result, the thickness of the photosensitive layer significantly influences photodetector performance. To standardize the Ga_2_O_3_ film thickness, the growth rate of Ga_2_O_3_ films on commercial FTO substrates was studied. The RF power, argon flow, and sputtering time were kept constant, while the oxygen flow was varied at 0.0, 0.2, 0.4, 0.6, 0.8, and 1.0 sccm during Ga_2_O_3_ thin film deposition. **Figures** **1**a and S1 (Supporting Information) show the cross‐sectional scanning electron microscopy (SEM) images of the Ga_2_O_3_ films under different oxygen flow conditions. Each sample exhibits a clear layered structure, with the Ga_2_O_3_ film, FTO conductive layer, and glass substrate identified through elemental line scans (Figure 1b). As shown in Figure S2 (Supporting Information), the Ga_2_O_3_ film thickness gradually decreases with increasing oxygen flow. Figure 1c illustrates the linear decrease in the growth rate with increasing oxygen flow, attributed to oxygen ion scattering.^[^ ^39^ ^]^ Based on this growth rate, the Ga_2_O_3_ film thickness was standardized, with sputtering time parameters under different oxygen flows provided in Figure S3 (Supporting Information). The prepared samples were labeled S0.0, S0.2, S0.4, S0.6, S0.8, and S1.0 according to the respective oxygen flow conditions.

Figure 1d,e presents the X‐ray diffraction (XRD) patterns and Raman scattering spectra for the Ga_2_O_3_ film (S0.0), respectively. All observed diffraction peaks and vibrational modes are attributed to the substrates, with no peaks corresponding to the crystalline structure of Ga_2_O_3_ detected, confirming that the Ga_2_O_3_ thin films are amorphous.^[^ ^27^ ^]^ This observation is consistent with the behavior of Ga_2_O_3_ films deposited by magnetron sputtering at room temperature.^[^ ^40^ ^]^ Additionally, Figures S4 and S5 (Supporting Information) provide information on films prepared under varying oxygen flow, revealing that changes in oxygen flow did not alter the amorphous state of the Ga_2_O_3_ films. Figure 1f demonstrates the relationship between (αhν)^2^ and photon energy (hν) for amorphous Ga_2_O_3_ thin films deposited on sapphire substrates under different oxygen flow conditions, yielding a consistent bandgap of 4.62 ± 0.03 eV. This finding is consistent with Mei et al.,’s work.^[^ ^41^ ^]^ To further determine the effect of oxygen flow on the oxygen vacancy (V_O_) concentration of amorphous Ga_2_O_3_ films, X‐ray photoelectron spectroscopy (XPS) analyses were conducted on specimens S0.0, S0.6, and S1.0. Figure S6 (Supporting Information) and Figure 1g present the Ga 2p core‐level spectra of these specimens, revealing a shift in Ga 2p_3/2_ binding energy toward higher values (from 1118.04 to 1118.27 eV) with increasing oxygen flow. This shift indirectly suggests a gradual reduction in oxygen vacancy concentration within the a‐Ga_2_O_3_ thin films.^[^ ^37^, ^38^, ^39^, ^40^, ^41^ ^]^ Figure 1h displays the Electron paramagnetic resonance (EPR) spectra of a‐Ga_2_O_3_ thin films, showing EPR signals at g = 2.003 in specimens S0.0, S0.6, and S1.0. This signal is commonly observed in oxide semiconductors and is often attributed to oxygen vacancies.^[^ ^42^, ^43^, ^44^, ^45^ ^]^ For instance, Zhang et al.^[^ ^45^ ^]^ observed EPR signals related to oxygen vacancies at g = 2.003 in WO_3_ materials, finding that the signal intensity increased with higher oxygen vacancy concentrations upon amorphization of WO_3_. In this study, the EPR signal was also observed in amorphous Ga_2_O_3_, and the intensity of these signals decreases with increasing oxygen flow (see Figure 1i). This observation confirms that the concentration of oxygen vacancies decreases with higher oxygen flow, consistent with the XPS results and previous reports.^[^ ^37^, ^38^, ^39^, ^40^, ^41^ ^]^

### 2.2 Performance Evaluation of Amorphous Ga_2_O_3_ PEC‐PDs

**Figure 2:**
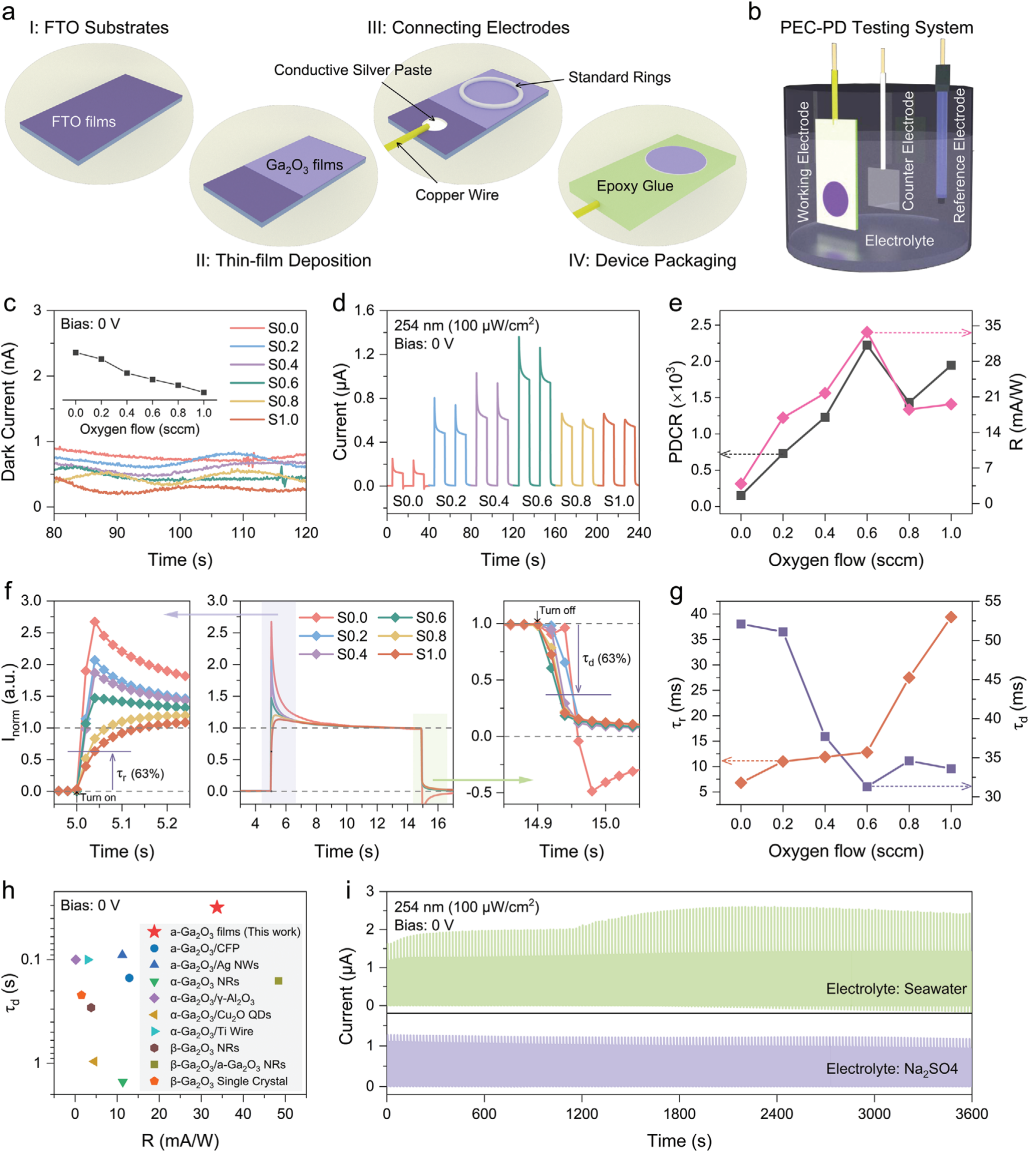
Self‐Powered Photodetection Performance of the PEC‐PDs. a) Schematic illustration of the fabrication process for a‐Ga_2_O_3_ film photoanodes. b) Schematic diagram of a typical PEC system used for evaluating the photoresponse behaviors of the a‐Ga_2_O_3_ PEC‐PDs. c) Dark currents and d) photoresponse behavior of a‐Ga_2_O_3_ PEC‐PDs with different oxygen flow. e) PDCR and responsivity as functions of oxygen flow at 0 V. f) *i–t* curves normalized to the steady‐state photocurrent. g) τ_r_ and τ_d_ as functions of oxygen flow. h) Comparison of the characterization parameters of self‐powered a‐Ga_2_O_3_ PEC‐PDs with other Ga_2_O_3_‐based PEC‐PDs. Detailed comparisons are listed in Table S1, (Supporting Information). i) Long‐term stability tests in Na_2_SO_4_ electrolyte and seawater (sourced from Bohai Bay).

**Figure** **2**a illustrates the preparation process for amorphous Ga_2_O_3_ PEC‐PDs, detailed in the experimental section. To comprehensively assess their photoelectric performance, a photoelectrochemical test system was set up (Figure 2b). The amorphous Ga_2_O_3_ thin film served as the photoanode, with a platinum (Pt) plate as the counter electrode and a saturated calomel electrode (SCE) as the reference electrode. The electrolyte used was a 0.5 m sodium sulfate (Na_2_SO_4_) aqueous solution. The self‐powered characterization principle based on PEC‐PDs is outlined in Figure S4 (Supporting Information). Electrical signals were recorded without applying a bias voltage using an electrochemical workstation, enabling the development of amorphous Ga_2_O_3_ thin film photoelectrochemical‐type solar‐blind UV‐C photodetectors. Two key parameters for evaluating photodetector performance are the photo‐dark current ratio (PDCR) and responsivity (R),^[^ ^46^ ^]^ calculated as follows:

(1) $$\mathrm{PDCR}=\frac{I_{\mathrm{photo}}}{I_{\mathrm{dark}}}$$

(2) $$R=\frac{I_{\mathrm{photo}}}{P_{\mathrm{inc}}S}$$

where I_photo_ = I_light_  − I_dark_ P_inc_ is the incident light intensity, and S is the effective illumination area of the device (0.2827 cm^2^). PDCR and R depend on the dark current in the device's dark state and the photocurrent under illumination. Figure S5 (Supporting Information) displays the current–time (*i–t*) curves of the PEC‐PDs under 0 V bias for dark conditions, showing the dark current characteristics. The average current of the last 30 s of a 120‐s test period represents the device's dark current. Figure 2c indicates that increasing oxygen flow reduces the dark current, attributed to decreased oxygen vacancy concentration. Oxygen vacancies typically act as donor defects that enhance carrier concentration and conductivity in metal‐oxide semiconductors like TiO_2_,^[^ ^47^ ^]^ WO_3_,^[^ ^48^ ^]^ and Fe_2_O_3_.^[^ ^49^ ^]^ Han et al. observed low dark current in amorphous Ga_2_O_3_‐based solid‐state devices due to high‐resistance film components.^[^ ^50^ ^]^ Figure 2d depicts *i–t* curves of the PEC‐PDs under periodic 20‐s light irradiation. All amorphous Ga_2_O_3_ thin‐film PEC‐PDs exhibit notable photoresponsive switching behavior at 0 V bias under 254 nm LED light (100 µW/cm^2^). Interestingly, Figure S6 (Supporting Information) shows that photocurrent initially increases and then decreases with rising oxygen flow. Figure 2e illustrates the variations in PDCR and R with oxygen flow, highlighting a peak R value of 33.75 mA W^−1^ at an oxygen flow of 0.6 sccm.

The response speed is one of the core metrics determining the performance of a photodetector. Figure 2f presents the *i–t* curves normalized by the stabilized photocurrent value to compare the response speed of each device. S0.0 and S0.2 exhibit noticeable persistent photoconductivity (PPC effect). The rise time (t_r_) is defined as the time for the photocurrent to increase from 0 to 63% (i.e., 1‐1/e), and the decay time (t_d_) is the time for the photocurrent to decrease from the maximum value to 37% (i.e., 1/e).^[^ ^51^ ^]^ Figure 2g plots t_r_​ versus td as a function of oxygen flow. The t_r_ of amorphous Ga_2_O_3_ thin film PEC‐PDs gradually increases with increasing oxygen flow, while td shows a gradual decrease. At an oxygen flow of 0.6 sccm, the devices exhibit a fast response with τ_r_/τ_d_ as fast as 12.8 ms/31.3 ms.

Encouragingly, the simultaneous enhancement of responsivity and response speed at an oxygen flow of 0.6 sccm, compared to the case with no oxygen flow, indicates that the appropriate amount of oxygen flow optimizes both parameters. Additionally, compared with most α‐Ga_2_O_3_, β‐Ga_2_O_3_, and amorphous Ga_2_O_3_‐based PEC‐PDs (see Figure 2h; Table S1, Supporting Information), the devices in this work exhibit higher responsivity and faster response speed. Figure S7 (Supporting Information) shows the spectral response curve of S0.6, which demonstrates a selective response to the UV‐C band. Figure 2i presents the *i–t* curves of S0.6 in Na_2_SO_4_ electrolyte and seawater (from Bohai Bay) for 1 h of continuous operation. The specimen maintains a good switching light response and excellent operational stability in both environments. There is a slight increase in photocurrent in seawater compared to Na_2_SO_4_ electrolyte, likely due to the complex ionic environment of seawater promoting redox product cycling. Nonetheless, the change in steady‐state photocurrent is nearly negligible, and Figure S11 (Supporting Information) demonstrates the strong corrosion resistance of the surface of the amorphous Ga_2_O_3_ films before and after long‐term testing, suggesting that the device has the potential to operate in seawater environments.

### 2.3 Mechanisms for Enhancing Amorphous Ga_2_O_3_ PEC‐PD Performance

The photoelectric conversion process in PEC‐PDs primarily occurs at the semiconductor/electrolyte interface (S–E interface).^[^ ^52^, ^53^ ^]^ This includes electrocatalytic reactions in the Helmholtz layer and the photovoltaic effect (compensating for the required input bias) in the neighboring space‐charge layer.^[^ ^54^ ^]^ By decoupling the PEC processes into electrochemical (EC) and photovoltaic (PV) components, the impact of oxygen vacancies (V_O_) on amorphous Ga_2_O_3_ PEC‐PDs can be clearly understood. Since this study does not involve modification with noble metal^[^ ^55^ ^]^ or transition metal oxide^[^ ^56^ ^]^ co‐catalysts, the EC process is mainly attributed to active sites such as V_O_ defects.^[^ ^54^, ^57^, ^58^ ^]^ Previous analysis shows that the V_O_ concentration in amorphous Ga_2_O_3_ films decreases with increasing oxygen flow, suggesting that the EC process weakens and does not enhance the performance of the PEC‐PDs.

**Figure 3:**
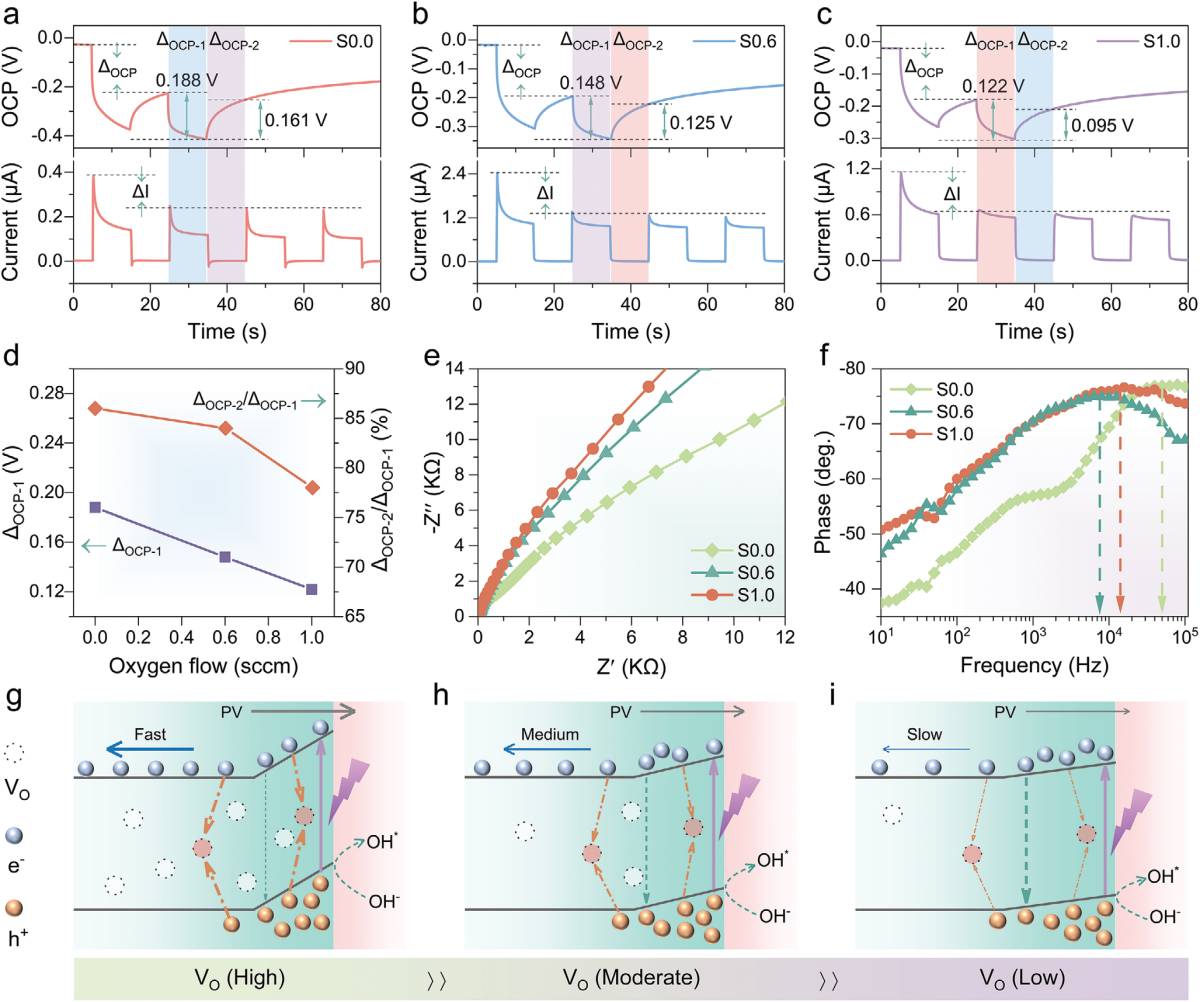
Charge‐Transfer Properties of PEC‐PDs. a–c) Photovoltage and photocurrent analyses of S0.0, S0.6, and S1.0. d) Δ_OCP‐1_ and Δ_OCP‐2_/Δ_OCP‐1_ of different devices. e,f) Nyquist plots and Bode phase curves of S0.0, S0.6, and S1.0. g) Schematic diagram of charge transfer in self‐powered a‐Ga_2_O_3_ PEC‐PDs with different V_O_ defects.

To investigate the carrier transport behavior mediated by V_O_ defects under light conditions, photovoltages at S0.0, S0.6, and S1.0 were tested using open‐circuit potential (OCP) measurements (see **Figure** **3**a–c). The results show that none of the devices could return to the initial OCP state after the first light cycle, with a noted difference (Δ_OCP_), and the OCP experienced a long relaxation period after periodic light exposure (Figure S8, Supporting Information). Corresponding *i–t* curves showed a significant photocurrent during the first illumination, followed by relatively smaller and stable photocurrents in subsequent cycles (Figure S9, Supporting Information), all with a decrease in ΔI. This indicates a significant correlation between Δ_OCP_ and ΔI during the first illumination, likely due to surface adsorption on the photoanode or trapping of photocarriers by defective states.^[^ ^59^ ^]^ To avoid the effects of the initial cycle on device performance, subsequent stable cycles were used to evaluate photocurrent and OCP parameters. Based on OCP test results, V_O_ defect‐mediated carrier transport behavior is considered from two aspects. First, Δ_OCP‐1_ reflects the light‐induced PV process, affecting the separation of photogenerated carriers.^[^ ^60^ ^]^ The Δ_OCP‐1_ values for S0.0, S0.6, and S1.0 are 0.188, 0.148, and 0.122 V, respectively, with Figure 3d showing a linear decrease in Δ_OCP‐1_ as V_O_ concentration increases. This indicates a weakening PV effect and photovoltage. Second, the recovery rate (Δ_OCP‐2_/Δ_OCP‐1_) responds to defect‐mediated indirect recombination.^[^ ^61^ ^]^ Figure 3d shows Δ_OCP‐2_/Δ_OCP‐1_ as a function of oxygen flow: 86%, 84%, and 78% as V_O_ concentration decreases. This indicates that the V_O_‐mediated carrier recombination rate gradually decreases with decreasing V_O_ concentration.

Figure 3e presents the electrochemical impedance spectra (EIS), where the charge transfer resistance (R_ct_) is indicated by the diameter of the Nyquist plot.^[^ ^62^ ^]^ As the V_O_ concentration decreases, the charge transfer resistance at the S–E interface increases, suggesting that a higher V_O_ concentration facilitates charge transfer. The Porter phase diagram, which effectively characterizes carrier lifetime, shows that a lower maximum frequency (f_max_) implies a longer carrier lifetime.^[^ ^63^ ^]^ Figure 3f demonstrates that the fmax values for S0.0, S0.6, and S1.0 are 79433, 6310, and 15849 Hz, respectively. This indicates that carrier lifetime initially increases and then decreases with decreasing V_O_ concentration, with S0.6 having the longest carrier lifetime. In summary, both surface catalytic and charge transfer capabilities weaken as V_O_ concentration decreases, which negatively impacts device performance. However, the weakening of V_O_ defect‐mediated indirect recombination is beneficial. These combined effects result in an initial increase and then a decrease in carrier lifetime, aligning with the observed photocurrent behavior. Figure 3g–i illustrates the carrier dynamics mechanism with decreasing V_O_ concentration: 1) Without additional oxygen (see Figure 3g), the amorphous Ga_2_O_3_ film has a high V_O_ concentration, enhancing catalytic reactions and charge transfer due to high conductivity, high photovoltage, and low interfacial transfer resistance. However, V_O_ defect‐mediated indirect recombination significantly reduces carrier lifetime and responsivity. Accumulation of interfacial holes exacerbates recombination and can lead to diffusion into the bulk phase, further increasing the recombination probability. 2) With moderate oxygen flow (see Figure 3h), V_O_ concentration is reduced, significantly decreasing indirect recombination and prolonging carrier lifetime, thereby improving responsivity, despite some loss in charge transfer capability. 3) With excessive oxygen flow (see Figure 3i), the V_O_ concentration is greatly reduced, leading to weakened transfer ability and surface catalysis. Photocarriers that cannot be transferred accumulate on the surface, intensifying direct electron–hole recombination and reducing carrier lifetime and responsivity. Thus, an appropriate oxygen flow can balance V_O_‐mediated indirect recombination, charge transfer, and surface catalytic reactions, enhancing the responsivity of amorphous Ga_2_O_3_ thin film PEC‐PDs.

For the response speed, the increase in τ_r_ with higher oxygen flow is due to a gradual decrease in the PV effect at the solid‐liquid interface and an increase in transfer resistance. Additionally, V_O_ not only impacts conductivity and carrier recombination in amorphous Ga_2_O_3_ films but also acts as a trap for photocarriers, which are slowly released when the light is turned off, leading to the PPC effect. Appropriate oxygen flow optimizes V_O_ concentration, suppressing the PPC effect and reducing τ_d_, thereby improving response speed. Balancing oxygen flow in amorphous Ga_2_O_3_ thin film PEC‐PDs allows for the simultaneous optimization of device responsivity and response speed by mitigating detrimental factors like carrier recombination and the PPC effect caused by oxygen vacancies. However, since oxygen vacancies also enhance charge transfer and surface catalysis, careful engineering of oxygen vacancies is essential to maximize overall device performance.

### 2.4 Mechanisms Behind Transient Currents in Amorphous Ga_2_O_3_ PEC‐PDs

**Figure 4:**
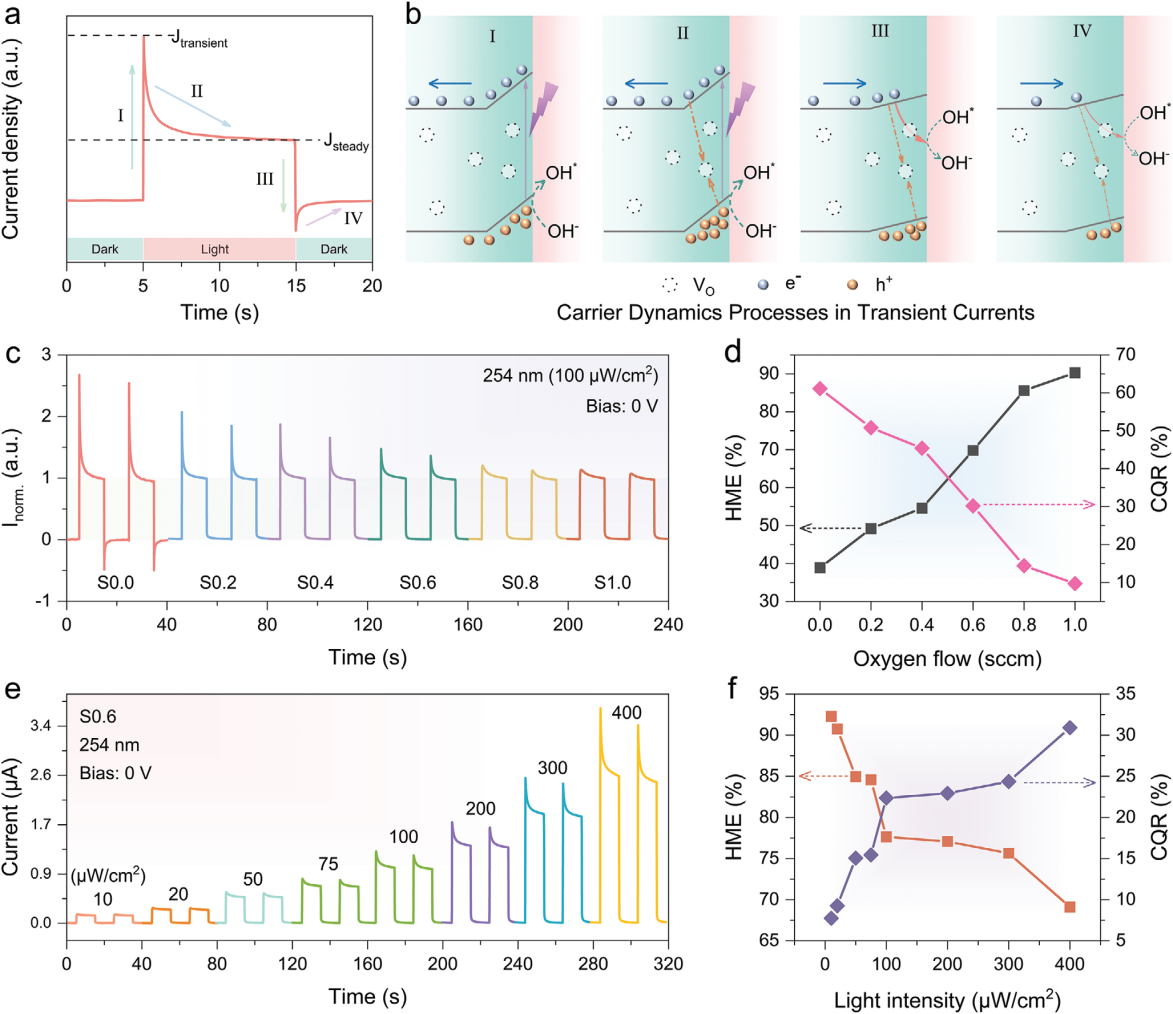
Mechanism of Transient Spike Currents. a) Typical transient photocurrent behavior and b) schematic representation of carrier dynamics in transient currents of a‐Ga_2_O_3_ PEC‐PDs. c) *i–t* curves normalized to the steady‐state photocurrent. d) HME and CQR as functions of oxygen flow. e) Photoresponse of S0.6 under illumination at 254 nm with various light power intensities from 10 to 400 µW cm^−2^. (f) HME and CQR of S0.6 as functions of light power intensities.

Transient currents in PEC‐PDs have raised significant concerns. Apparent transient current spikes are observed in the *i–t* curves in Figure 3a–c. **Figure** **4**a shows the *i–t* curves of a single cycle of a typical PEC‐PD, highlighting pronounced transient currents. The cycle can be divided into four processes: I) When the light is turned on, the current rises from the dark state to the photocurrent peak (J_transient_). II) With continuous illumination, the photocurrent decreases from the peak (J_transient_) to a stable value (J_steady_). III) After turning off the light, the current drops rapidly, and a reverse overshoot occurs.

Based on previous reports and experimental results,^[^ ^64^, ^65^ ^]^ the carrier dynamics of the transient current in the amorphous Ga_2_O_3_ photoanode are depicted in Figure 4 b: I) The transient photocurrent (J_transient_) observed when the light is turned on corresponds to the rapid separation of photogenerated electron–hole pairs in the space charge region. Holes move to the interface while electrons are transported to the external circuit via the n‐type amorphous Ga_2_O_3_. II) The subsequent rapid decay of the photocurrent is due to the accumulation of photogenerated holes at or near the interface, leading to interfacial recombination and a decrease in photocurrent. After a period of sustained light, the photocurrent reaches a steady state (J_steady_) where the rate of photogenerated holes reaching the interface balances with the rate of transfer and recombination, resulting in a constant photocurrent across the semiconductor/electrolyte interface. III) When the light is turned off, the interfacial photovoltage disappears instantly. Surface defect states assist electrons in the bulk phase to cross the space charge region in reverse, reaching the electrolyte and recombining with the remaining holes on the surface. IV) In sustained darkness, the reverse current gradually decays until all excess electrons are consumed by recombination and charge transfer, eventually returning the current to its initial dark state value.

To visually compare the changing pattern of transient current due to oxygen flow, Figure 4c shows the *i–t* curve normalized by the steady‐state current (J_steady_). A clear decreasing trend in the transient photocurrent spike is observed with increasing oxygen flow. This indicates that carrier recombination in the transient photocurrent is dominated by V_O_ concentration. The efficiency of photocarrier utilization during the I‐II process is studied by the ratio of J_steady_ to J_transient_, which denotes the hole migration efficiency (HME).^[^ ^66^ ^]^ The carrier quenching rate (CQR) is defined as 1 – HME to represent the recombination ratio of photogenerated carriers due to surface hole accumulation. Figure 4d shows the variation patterns of HME and CQR with oxygen flow. The HME rate gradually increases, and the CQR decreases with increased oxygen flow, confirming that high V_O_ concentration leads to severe indirect recombination of photocarriers. This recombination can be suppressed by passing trace amounts of oxygen during sputtering. Additionally, a reverse current spike was observed in S0.0 (V_O_‐enriched) after turning off the light but disappeared in all other devices after passing oxygen, attributed to the suppression of V_O_‐assisted tunneling.

Figure 4e shows the *i–t* curves of the device S0.6 for different light irradiation intensities (10‐400 µW cm^−^^2^) at 0 V bias voltage. All photocurrents increase almost linearly with increasing optical power intensity, clearly distinguishing different light intensities. With increased light intensity, transient current spikes also show an obvious increasing trend. Figure 4f illustrates the variation of HME and CQR with light intensity. As light intensity increases, HME decreases and CQR increases. This suggests that higher optical power density generates more photogenerated carriers, exacerbating surface hole accumulation and increasing recombination probability.^[^ ^22^ ^]^ Meanwhile, Figure S10 (Supporting Information) demonstrates the relationship between R and light intensity, showing a gradual decrease with increasing light intensity, further proving that high light intensity exacerbates photogenerated carrier recombination.

### 2.5 Proof‐of‐Concept Demonstration of Solar‐Blind Underwater Imaging

**Figure 5:**
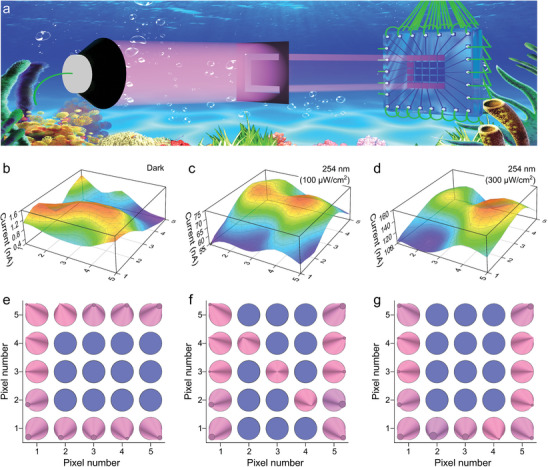
Proof‐of‐Concept Demonstration of Solar‐Blind Underwater Imaging. a) Schematic of the solar‐blind underwater imaging system with a‐Ga_2_O_3_ PEC‐PD arrays. b–d) 3D current mapping of a‐Ga_2_O_3_ PEC‐PD arrays irradiated in the dark, and 254 nm with 100 and 400 µW cm^−2^ light intensity, respectively. e,f) Underwater imaging based on a‐Ga_2_O_3_ PEC‐PD arrays, clearly distinguishing the shapes of the characters “C”, “N”, and “U”.

Underwater array imaging offers a significant speed advantage over single‐pixel imaging by rapidly capturing and processing image data, greatly improving the efficiency and accuracy of underwater detection.^[^ ^67^, ^68^ ^]^ The potential application of amorphous Ga_2_O_3_ thin‐film self‐powered PEC‐PD arrays for solar‐blind UV‐C underwater imaging was explored. Figure S11 (Supporting Information) outlines the fabrication process of the 5 × 5 matrix array imaging device using amorphous Ga_2_O_3_ thin‐film PEC‐PD (S0.6). **Figure** **5**a presents a schematic of the solar‐blind UV‐C underwater imaging system for amorphous Ga_2_O_3_ PEC‐PDs. Figure 5b shows a 3D image of the dark current for each pixel cell, which fluctuates weakly between 0.4 and 1.6 nA across 25 pixel points under 0 V bias, indicating high uniformity. Additionally, Figure 5c,d provide 3D images of the photocurrent of the PEC‐PD array under 254 nm illumination, with fluctuation intervals of 55–75 nA (100 µW cm^−^^2^) and 100–160 nA (300 µW/cm^2^), respectively, demonstrating good resolution. Figure 5e–g, along with Figure S12 (Supporting Information), show the results of underwater imaging using amorphous Ga_2_O_3_ PEC‐PD arrays, clearly distinguishing the shapes of the characters “C”, “N”, and “U”. These results indicate that amorphous Ga_2_O_3_ PEC‐PD arrays have preliminary solar‐blind UV‐C imaging capability, providing a reference for future applications of PEC‐PDs in solar‐blind UV‐C underwater imaging systems.

## 3 Conclusion

In conclusion, our study focused on optimizing self‐powered PEC‐PD arrays based on oxygen‐vacancy‐tuned a‐Ga_2_O_3_ thin films for solar‐blind underwater imaging applications. Through a one‐step sputtering process with controlled oxygen flow, we successfully fabricated a‐Ga_2_O_3_ thin films with varying V_O_ concentrations. This optimization aimed to enhance the responsivity and response speed of the PDs by balancing electrocatalytic reactions, charge transfer dynamics, and carrier recombination processes. Our optimized a‐Ga_2_O_3_‐based PEC‐PDs exhibited outstanding performance metrics, achieving a high responsivity of 33.75 mA W^−1^ and rapid response times of 12.8/31.3 ms. Moreover, we observed a significant correlation between anomalous transient photocurrent spikes and V_O_ defect concentrations, indicating V_O_‐mediated indirect recombination processes. Finally, our proof‐of‐concept solar‐blind underwater imaging system, employing these optimized PEC‐PDs, successfully demonstrated clear imaging capabilities in seawater. This research underscores the potential of cost‐effective and high‐performance a‐Ga_2_O_3_ thin‐film‐based PEC‐PDs for advancing solar‐blind underwater imaging technology, offering promising prospects for future practical applications in marine exploration and reconnaissance.

## 4 Experimental Section

### Preparation and Characterization of a‐Ga_2_O_3_ Thin Films

The a‐Ga_2_O_3_ thin films were deposited on an FTO conductive substrate (10 mm × 20 mm) using RF magnetron sputtering at room temperature. The background vacuum was maintained at 5 × 10⁻⁴ Pa, the sputtering pressure at 1 Pa, and the RF power at 150 W. A mixed high‐purity argon and oxygen flow was used to control the oxygen vacancy concentration in the films. The argon flow was set at 40 sccm, while the oxygen flow was varied at 0.0, 0.2, 0.4, 0.6, 0.8, and 1.0 sccm. The growth rates of a‐Ga_2_O_3_ films at different oxygen flows were measured. To ensure consistent film thickness, the sputtering time was adjusted based on the growth rates, keeping the thickness of all films near 360 nm. The cross sectional micromorphology, elemental composition, and distribution maps were examined using a Thermo Scientific Helios 5 CX field emission scanning electron microscope (SEM). Structural analysis was performed using a Rigaku Ultima IV X‐ray diffractometer (XRD, Cu Kα rays: λ = 0.1540598 nm) and Raman scattering spectroscopy (Horiba HR Evolution, laser wavelength: λ = 532 nm). Absorption spectra were measured using a U4100 UV–Vis–NIR spectrophotometer. Elemental analysis was conducted with X‐ray photoelectron spectroscopy (XPS) using a monochromatic Al Kα source (ThermoFisher, ESCALAB 250Xi, USA), with binding energy calibrated to the C 1s peak at 284.6 eV. The oxygen vacancy concentration was analyzed using a Bruker Model A300 Electron Spin Resonance Spectrometer (ESR/EPR).

### Packaging and Testing of a‐Ga_2_O_3_ Thin‐Film PEC‐PDs

For individual PEC‐PDs, one end of a conductive copper rod was attached to the FTO substrate without sputtering a‐Ga_2_O_3_ thin film using silver conductive adhesive. A polytetrafluoroethylene standard ring (0.2827 cm^2^) was placed in the center of the a‐Ga_2_O_3_ film, coated with commercial epoxy AB adhesive (9462 s Hysol epoxy adhesive), and hardened in a vacuum to obtain a single a‐Ga_2_O_3_ thin‐film PEC‐PD. For arrays of a‐Ga_2_O_3_ thin‐film PEC‐PDs, a patterned 5 × 5 matrix FTO substrate (25 mm × 30 mm, with a single pixel cell area of 0.04 cm^2^) was used. The other sputtering and packaging processes remained consistent with those described above. The photoelectrochemical performance of the devices was evaluated in a self‐assembled three‐electrode system using a CHI440C electrochemical workstation. The packaged a‐Ga_2_O_3_ thin film specimens served as the working electrodes, with a saturated calomel electrode (SCE) as the reference electrode and a platinum sheet as the counter electrode. The electrolytes used were 0.5 m Na_2_SO_4_ aqueous solution and Bohai Bay seawater, and a commercial UV‐C LED served as the 254 nm light source. All measurements were conducted at room temperature.

## Conflict of Interest

The authors declare no conflict of interest.

## Supporting information



Supporting Information

## Data Availability

The data that support the findings of this study are available from the corresponding author upon reasonable request.
