# Supplementary material for: Balancing Carrier Dynamics in Oxygen‐Vacancy‐Tuned Amorphous Ga2O3 Thin‐Film Self‐Powered Photoelectrochemical‐Type Solar‐Blind Photodetector Arrays for Underwater Imaging
Source: Adv Sci (Weinh). 2024 Sep 30;11(43):2407822. doi: 10.1002/advs.202407822 (PMC11578302; doi:10.1002/advs.202407822)
Supplement: Supplementary file 1 — Supporting Information [file ADVS-11-2407822-s001.docx]

*Supporting Information*

**Balancing Carrier Dynamics in Oxygen-Vacancy-Tuned Amorphous Ga_2_O_3_ Thin-Film Self-Powered Photoelectrochemical-type Solar-Blind Photodetector Arrays for Underwater Imaging**

Ke Ding, Hong Zhang, Jili Jiang, Jiangshuai Luo, Rouling Wu, Lijuan Ye, Yan Tang, Di Pang, Honglin Li and Wanjun Li^*^

K. Ding, H. Zhang, J. L. Jiang, J. S. Luo, R. L. Wu, L. J. Ye, Y. Tang, D. Pang, H. L. Li and W. J. Li

Chongqing Key Laboratory of Photo-Electric Functional Materials and Laser Technology, College of Physics and Electronic Engineering, Chongqing Normal University, Chongqing, 401331, People's Republic of China

^*^E-mail: [liwj@cqnu.edu.cn](mailto:liwj@cqnu.edu.cn) (W. J. Li)


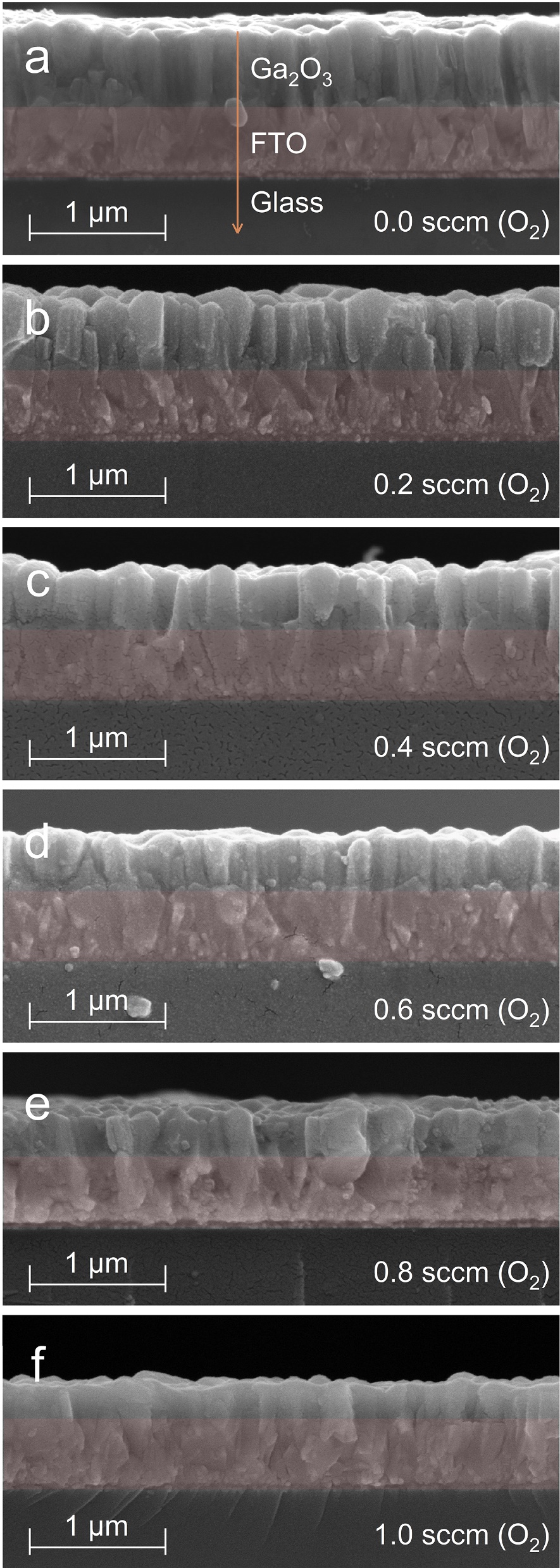


**Fig. S1.** Cross-sectional SEM images of the amorphous Ga_2_O_3_ thin films.

**
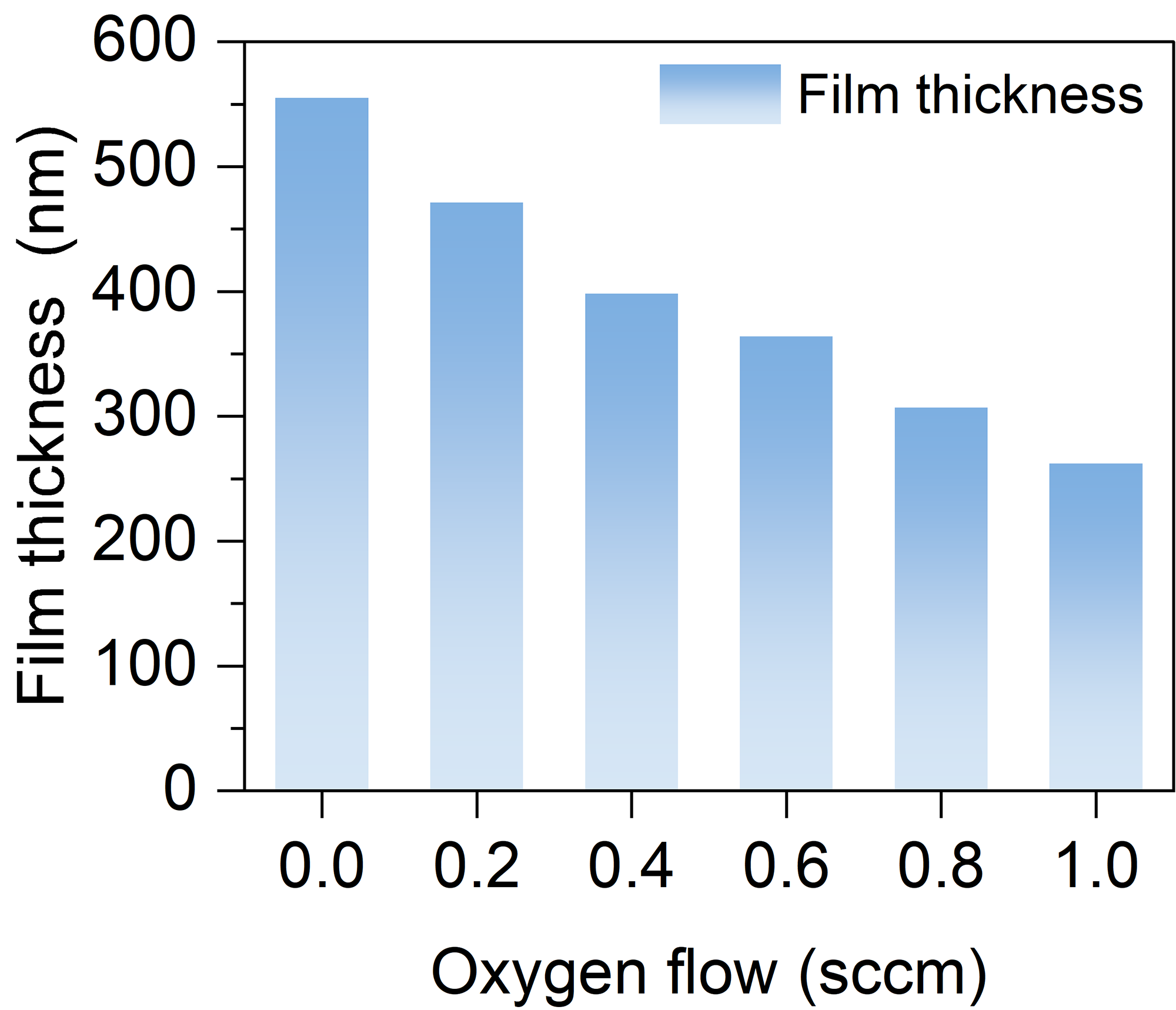
**

**Fig. S2.** Thickness of amorphous Ga_2_O_3_ thin films under varying oxygen flow.

**
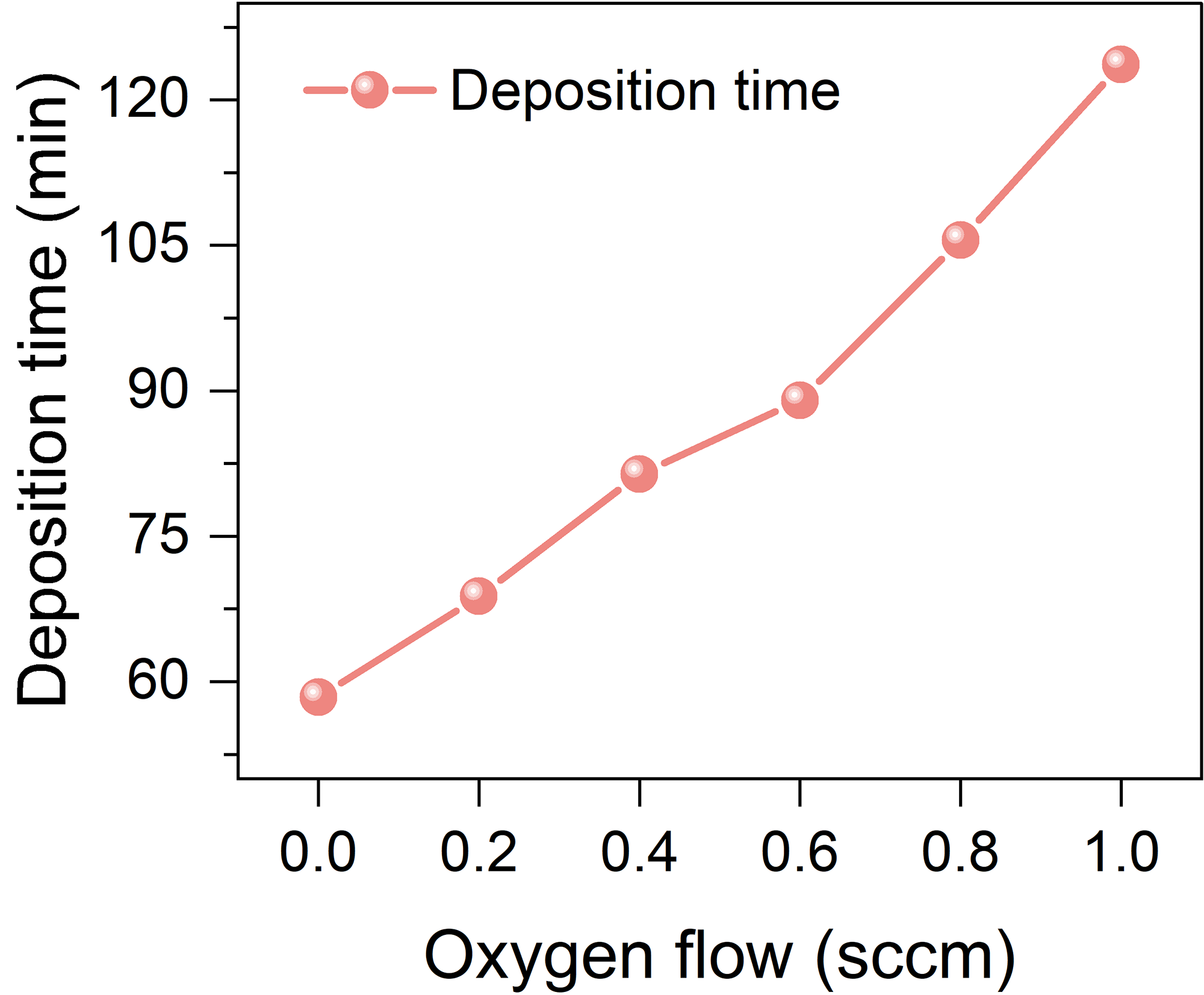
**

**Fig. S3.** Sputtering time corresponding to different oxygen flow for amorphous Ga_2_O_3_ thin films with a thickness set at ~360 nm.


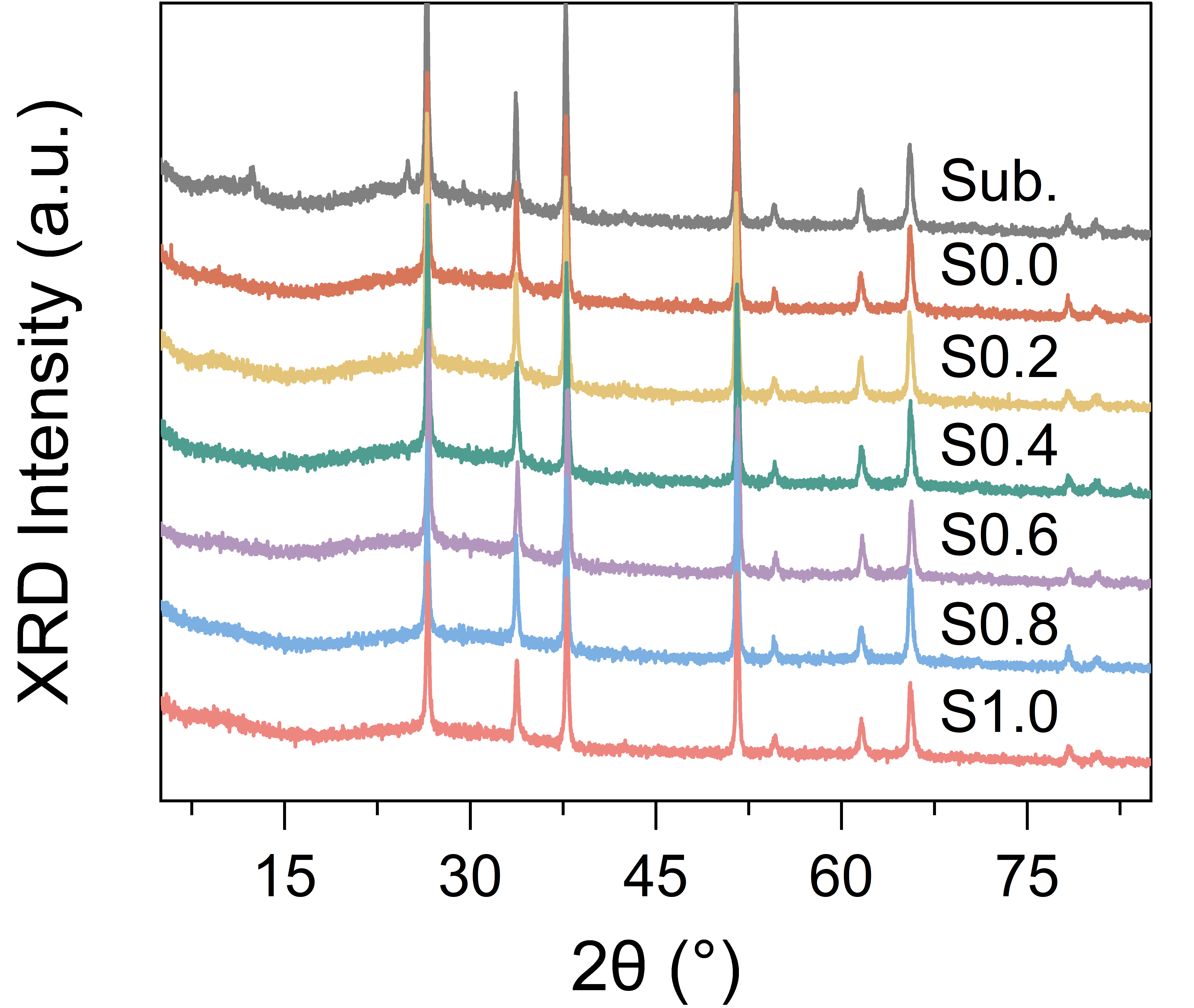


**Fig. S4.** XRD patterns of Ga_2_O_3_ films.


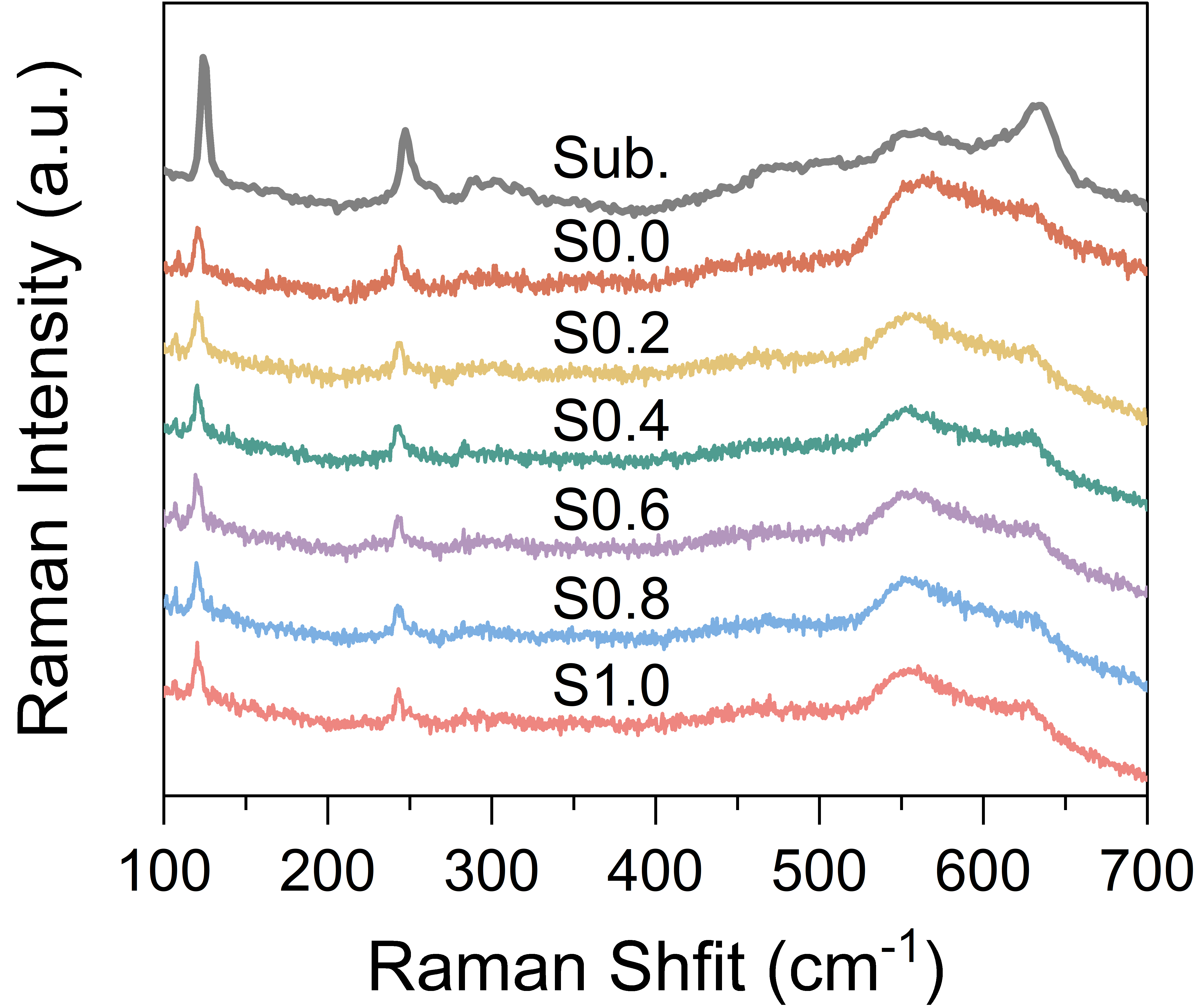


**Fig. S5.** Raman scattering spectra of Ga_2_O_3_ films.


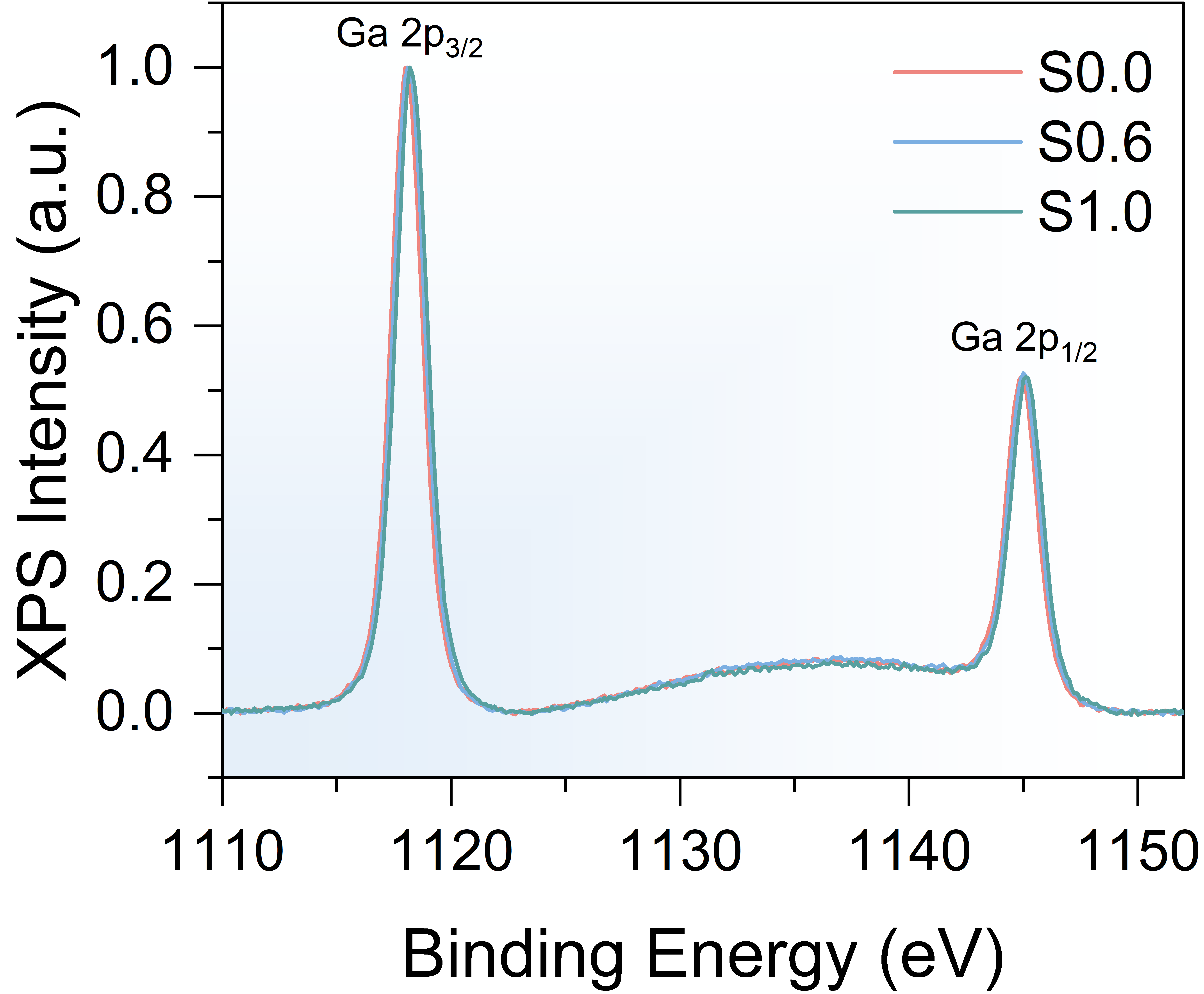


**Fig. S6.** XPS Ga 2p core-level spectra of S0.0, S0.6, and S1.0.

**
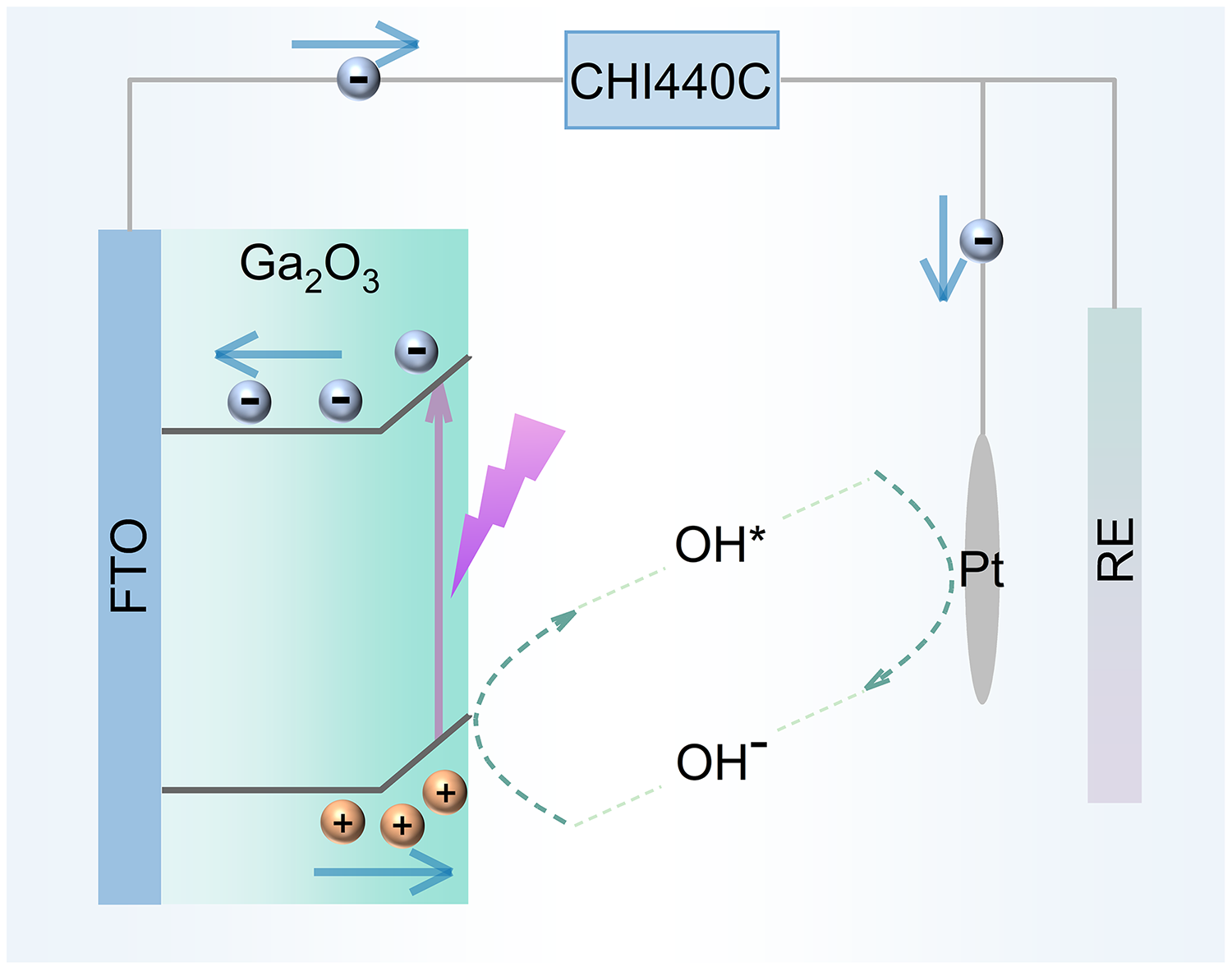
**

**Fig. S7.** Mechanism diagram of a self-powered photoelectrochemical-type photodetector (PEC-PD). When n-type amorphous Ga_2_O_3_ is in contact with the electrolyte, amorphous Ga_2_O_3_ exhibits upward energy band bending at the amorphous Ga_2_O_3_/electrolyte interface due to the establishment of electrochemical equilibrium. Upon deep ultraviolet (UV-C) light irradiation, electrons are excited from the valence band (E_V_) to the conduction band (E_C_), generating photogenerated electrons (e^-^) and holes (h^+^). The photogenerated electrons migrate to the semiconductor side and participate in the reduction reaction via an external circuit to the counter electrode, facilitated by an electric field within the space charge region. Photogenerated holes move to the amorphous Ga_2_O_3_/electrolyte interface, initiating an oxidation reaction. Reduction and oxidation products exchange substances in the electrolyte, forming a closed loop.


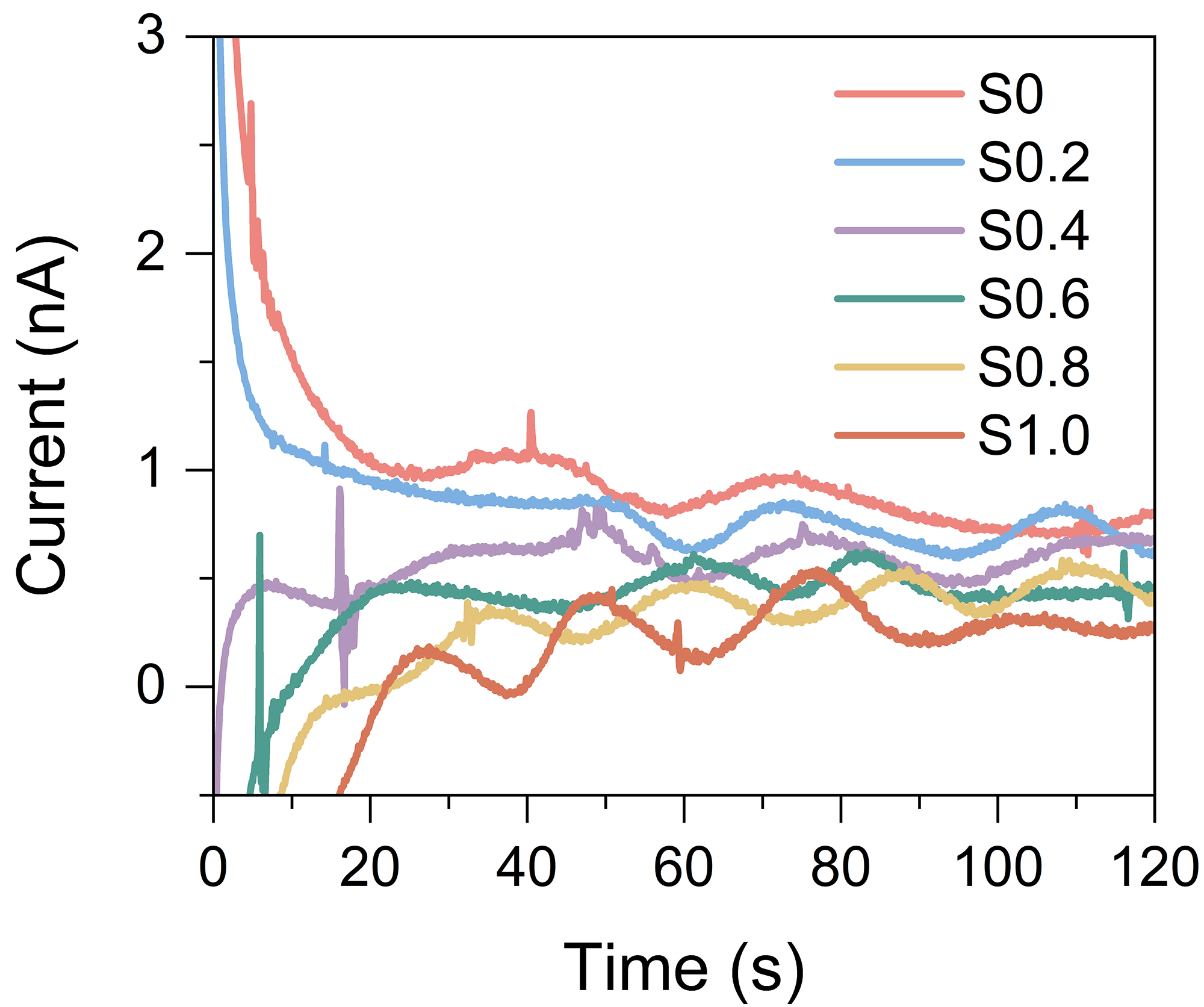


**Fig. S8.** Dark currents of amorphous Ga_2_O_3_ PEC-PDs with different oxygen flow.


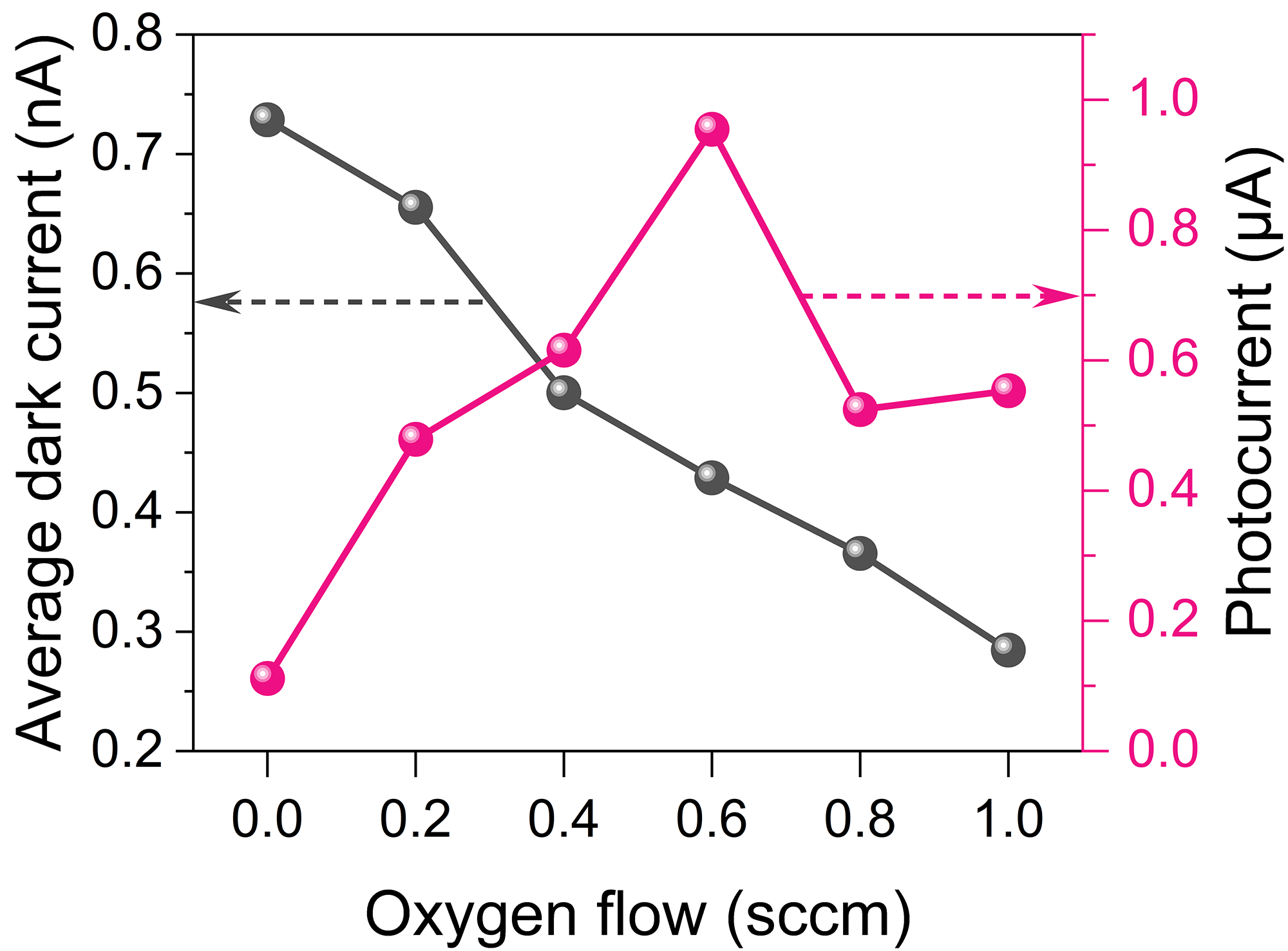


**Fig. S9.** Average dark current and photocurrent as functions of oxygen flow at 0 V.


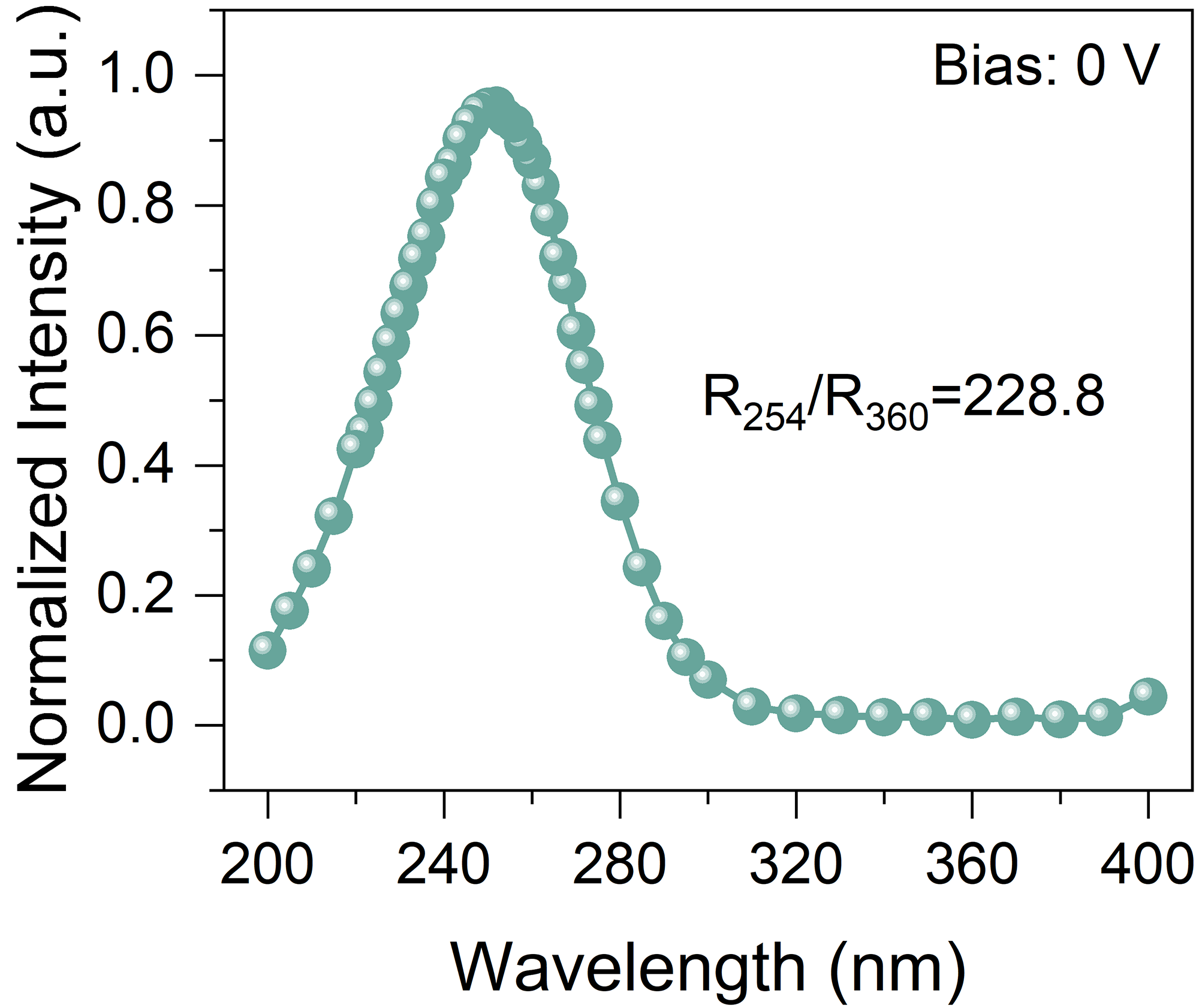


**Fig. S10.** Spectral response curve of amorphous Ga_2_O_3_ PEC-PD (S0.6) at 0 V.


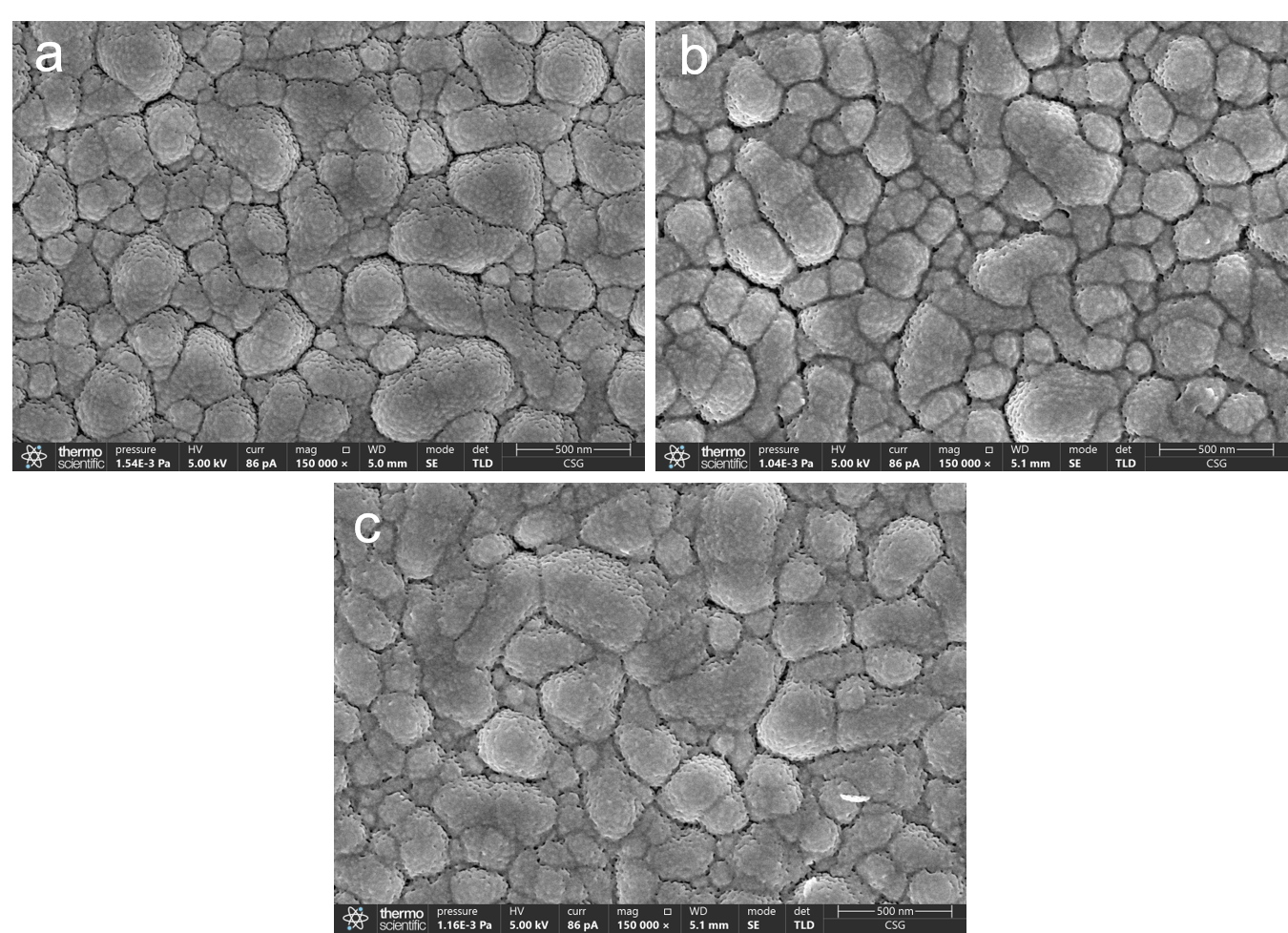


**Fig. S11.** SEM Images of amorphous Ga_2_O_3_ PEC-PD (S0.6) before and after testing. (a) & (b) SEM images of a freshly prepared device before and after a 1-hour prolonged test in Na_2_SO_4_ solution. (c) SEM image of the original device captured after undergoing cumulative testing for over 5 hours in both Na_2_SO_4_ solution and seawater, followed by more than 10 months of storage under ambient conditions. These demonstrate the strong corrosion resistance of the surface of the amorphous Ga_2_O_3_ films before and after long-term testing, suggesting that the device has the potential to operate in seawater environments.


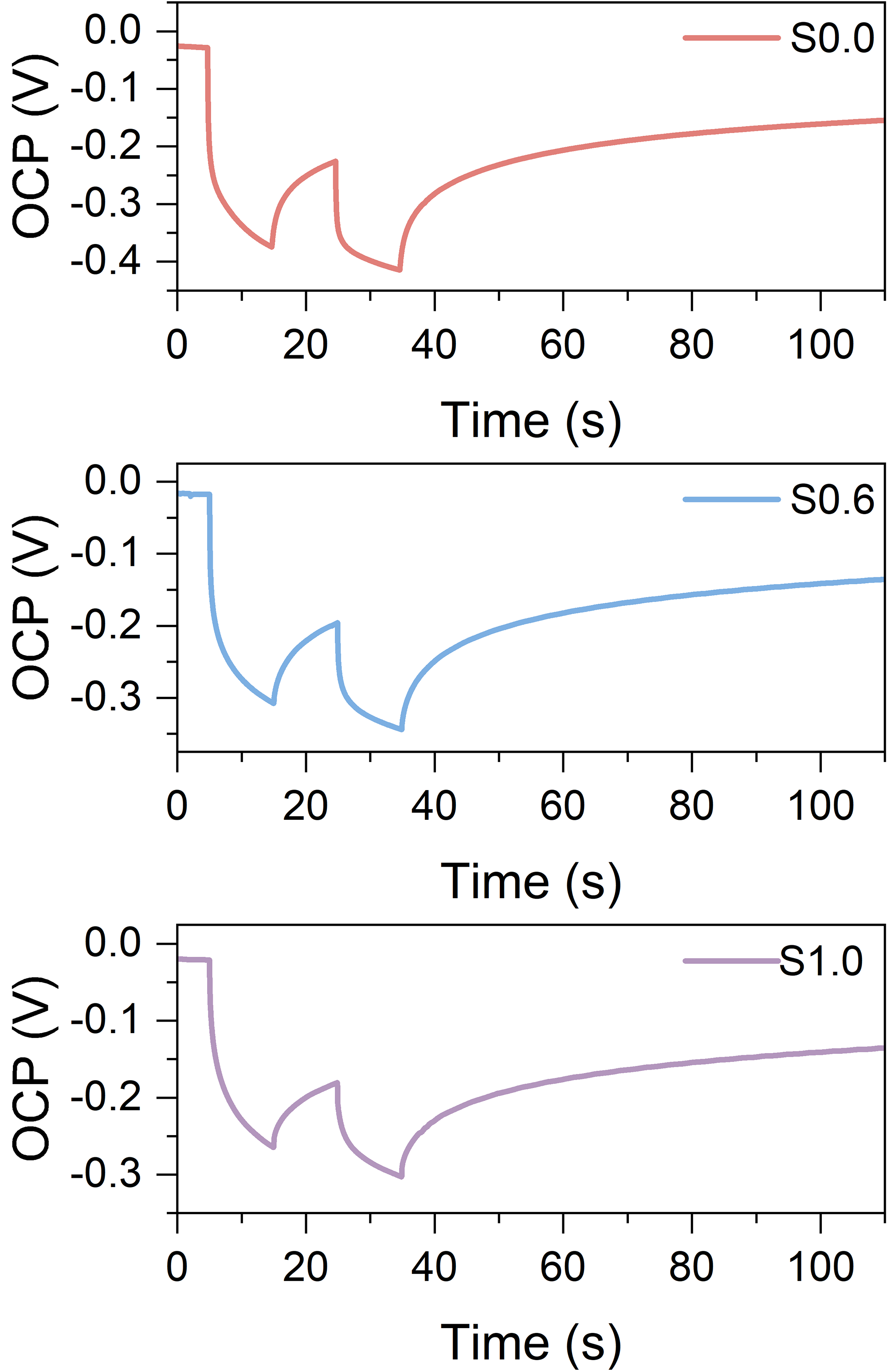


**Fig. S12.** Open circuit potential plots of S0.0, S0.6 and S1.0.


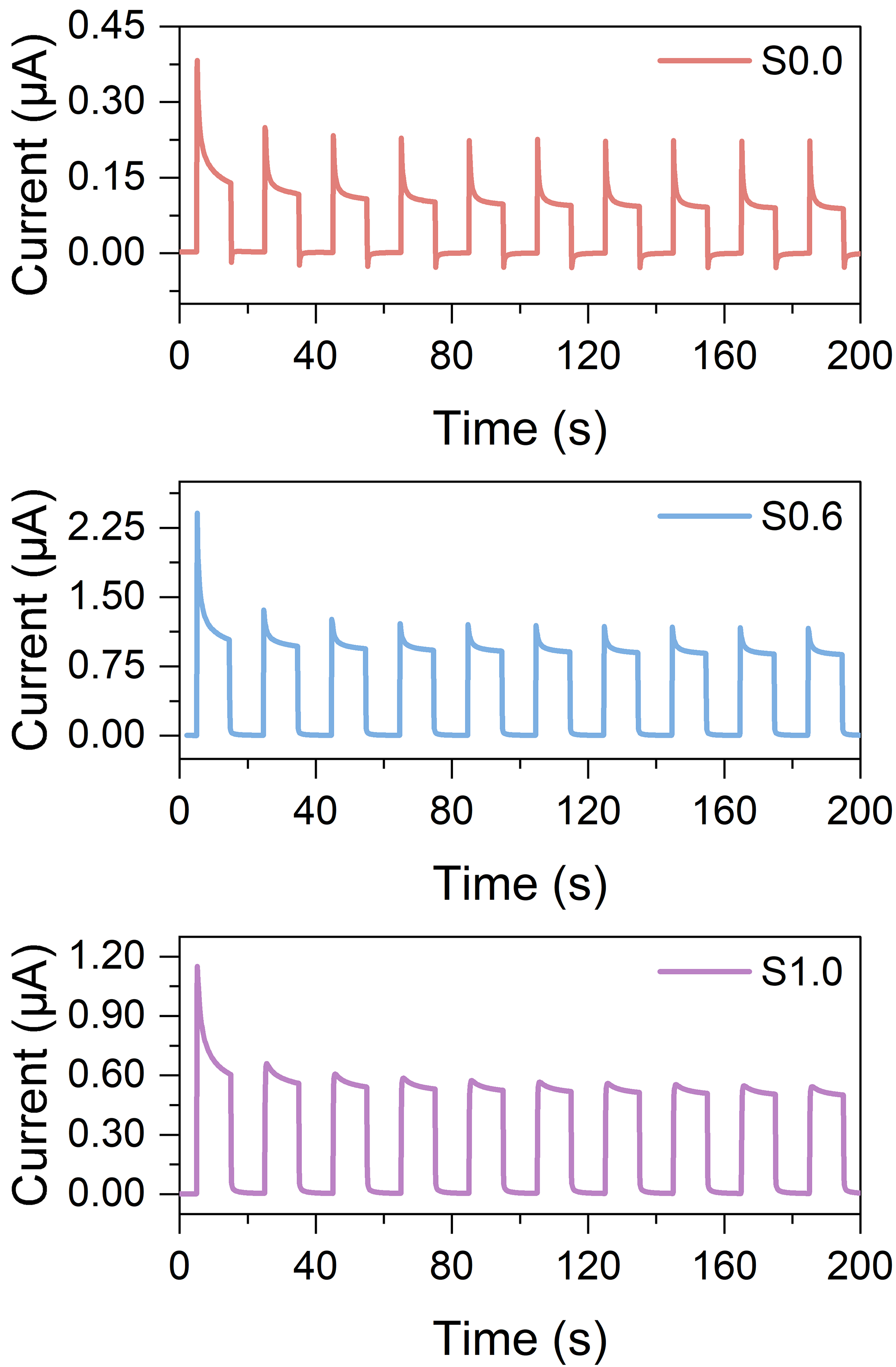


**Fig. S13.** I-t photoresponses of S0.0, S0.6 and S1.0.


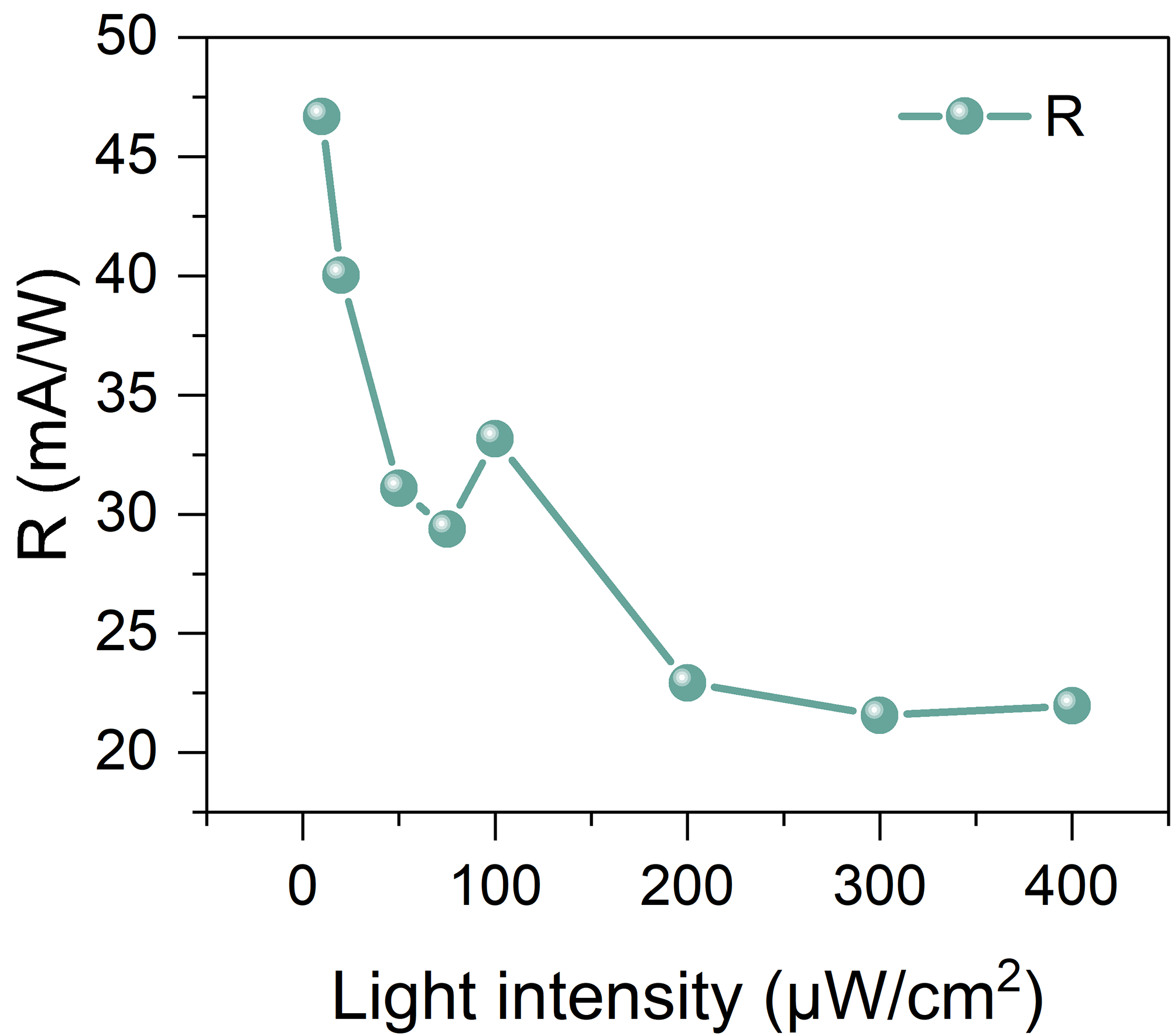


**Fig. S14.** Responsivity (R) of S0.6 under different light power intensities.


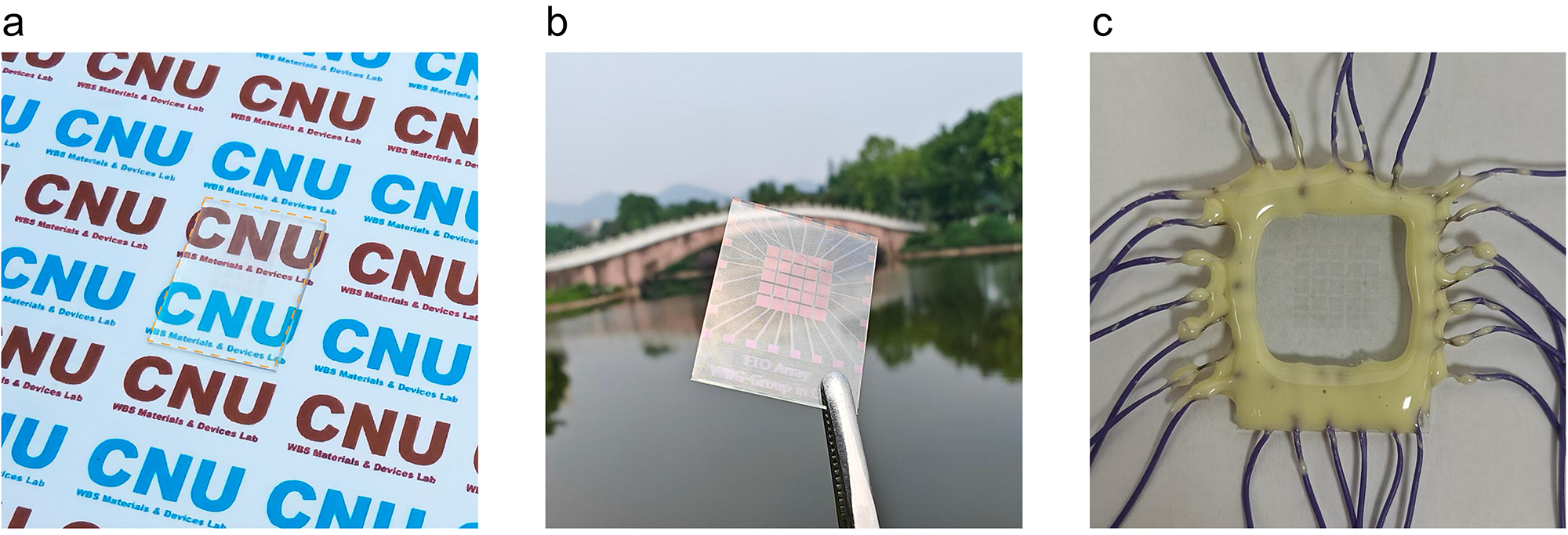


**Fig. S15.** (a-b) Photographs of a patterned 5×5 matrix FTO substrate (25 mm × 30 mm). (c) Packaged array of amorphous Ga_2_O_3_ Thin-Film PEC-PDs.


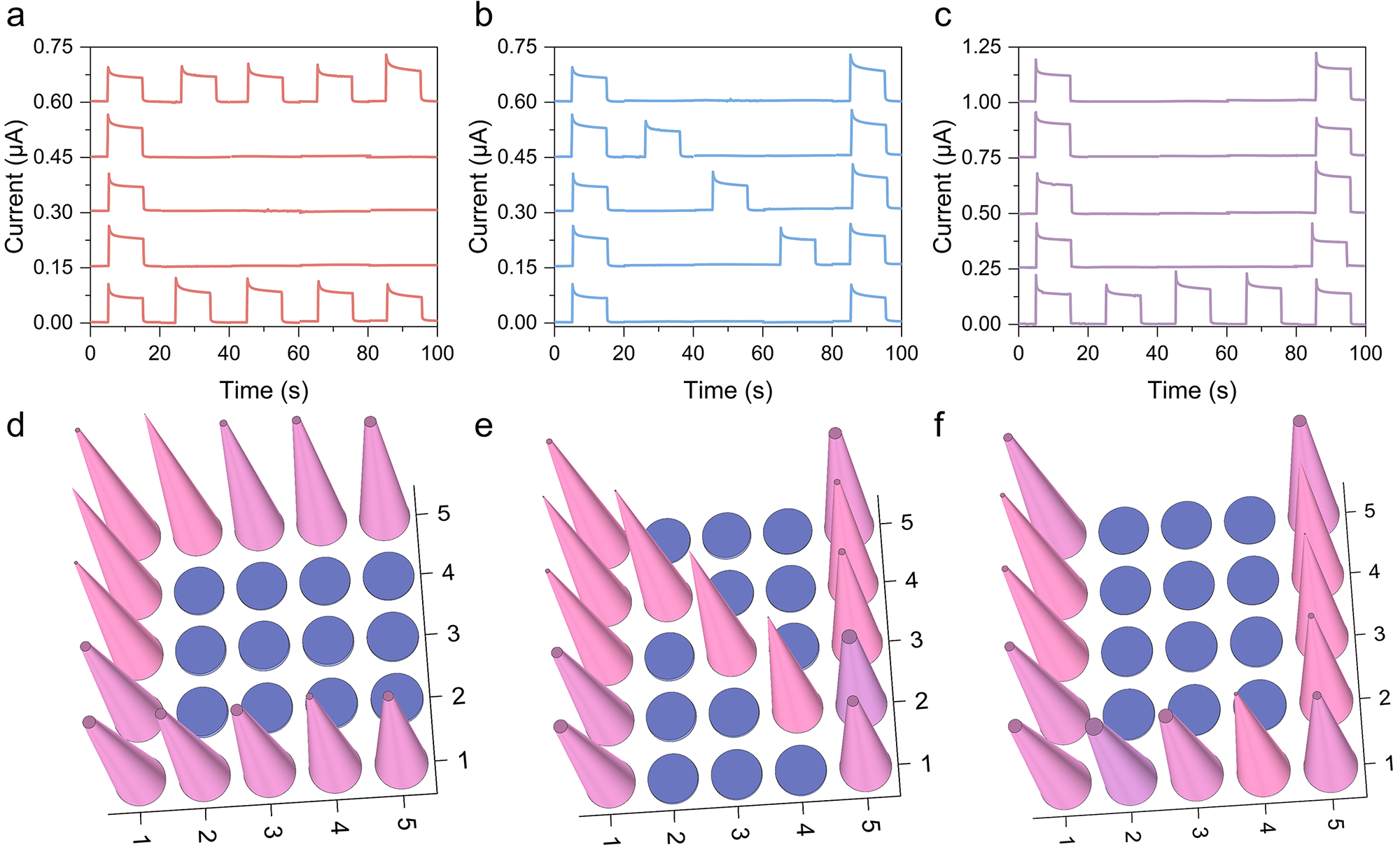


**Fig. S16.** (a-c) Raw data and (d-f) 3D maps of underwater imaging using arrays of amorphous Ga_2_O_3_ PEC-PDs.

**Supplementary Note 1: Density Functional Theory (DFT) Calculations:**

The DFT calculations, used to establish the amorphous Ga_2_O_3_ models, employed the Perdew-Berk-Ernzerhof (PBE) exchange-correlation functional with a cutoff energy of 500 eV for structural optimization. The computational models utilized a Monkhorst-Pack k-point grid of 5 × 5 × 1, a force threshold of 0.01 eV Å^−1^, and an energy convergence tolerance of 10⁻^4^ eV. The amorphous Ga_2_O_3_ structure was generated through molecular dynamics simulation, beginning with a β-Ga_2_O_3_ crystal. Different oxygen vacancy concentrations were first introduced in the β-Ga_2_O_3_ crystal, after which the system was rapidly heated from 0 to 3000 K over 0.3 ns under the NVT ensemble. This was followed by equilibration at 4000 K for 2.0 ns and a gradual cooldown to the melting point under the NPT ensemble, using a quenching rate of 1.0 K ps⁻¹.

**Table S1.** Comparison of characteristic parameters between amorphous Ga_2_O_3_ film self-powered PEC-PDs in this study and other previously reported Ga_2_O_3_-based solar-blind PEC-PDs.

| **PEC-PDs** | **Light condition** | **Electrode**  **type** | **Responsivity (mA/W)** | **Rise/decay time (s)** | **Refs.** |
| --- | --- | --- | --- | --- | --- |
| amorphous Ga_2_O_3_ films | 254nm (0.1 mW/cm^2^) | Na_2_SO_4_ | 33.75 | 0.0128/0.0313 | This work |
| amorphous Ga_2_O_3_/CFP | 254nm (0.1 mW/cm^2^) | Na_2_SO_4_ | 12.90 | 0.15/0.13 | [1] |
| amorphous Ga_2_O_3_@Ag NWs | 254nm (0.5 mW/cm^2^) | Na_2_SO_4_ | 11.23 | 0.07/0.09 | [2] |
| α-Ga_2_O_3_ NRs | 254 nm (0.5 mW/cm^2^) | Na_2_SO_4_ | 11.34 | 1.51/0.18 | [3] |
| α-Ga_2_O_3_/γ-Al_2_O_3_ | 254 nm (1.0 mW/cm^2^) | NaOH | 0.174 | 0.10/0.10 | [4] |
| α-Ga_2_O_3_/Cu_2_O QDs | 254 nm | Na_2_SO_4_ | 4.57 | 0.81/0.96 | [5] |
| α-Ga_2_O_3_ NRs/Ti wire | 254 nm (0.1 mW/cm^2^) | Na_2_SO_4_ | 3.0 | 0.1/0.06 | [6] |
| β-Ga_2_O_3_ NRs | 254 nm (2.8 mW/cm^2^) | Na_2_SO_4_ | 3.81 | 0.29/0.16 | [7] |
| β-Ga_2_O_3_NRs@a-Ga_2_O_3_ | 254nm (0.1 mW/cm^2^) | Na_2_SO_4_ | 48.39 | 0.125/0.16 | [8] |
| β-Ga_2_O_3_ single crystal | 213 nm | NaCl | 0.10 | 0.04/0.069 | [9] |

**References**

1. L. Huang, Z. Hu, H. Zhang, Y. Xiong, S. Fan, C. Kong, W. Li, L. Ye, H. Li, J. Mater. Chem. C. **2021**, 9, 10354.
2. C. Yu, H. Li, K. Ding, L. Huang, H. Zhang, D. Pang, Y. Xiong, P.-A. Yang, L. Fang, W. Li, Y. Tang, L. Ye, C. Kong, Adv. Opt. Mater. **2024**, 2400116.
3. L. Huang, Z. Hu, X. He, T. Ma, M. Li, H. Zhang, Y. Xiong, C. Kong, L. Ye, H. Li, W. Li, Optical Materials Express. **2021**, 11, 2089.
4. J. Zhang, S. Jiao, D. Wang, S. Gao, J. Wang, L. Zhao, Applied Surface Science.**2021**, 541, 148380.
5. P. Han, T. Kang, W. Chen, M. Gao, F. Teng, P. Hu, H. Fan, Journal of Alloys and Compounds.**2023**, 952, 170063.
6. Z. Chen, P. Han, W. Chen, Z. Wan, J. Yang, Z. Liu, P. Hu, F. Teng, H. Fan, ACS Applied Electronic Materials.**2023**, 6, 496.
7. K. Chen, S. Wang, C. He, H. Zhu, H. Zhao, D. Guo, Z. Chen, J. Shen, P. Li, A. Liu, C. Li, F. Wu, W. Tang, ACS Applied Nano Materials. **2019**, 2, 6169.
8. Y. Feng, L. Lv, H. Zhang, L. Ye, Y. Xiong, L. Fang, C. Kong, H. Li, W. Li, Appl. Surf. Sci. **2023**, 624, 157149.
9. N. Zhang, Z. Lin, Z. Wang, S. Zhu, D. Chen, H. Qi, W. Zheng, ACS Nano. **2023**, 18, 652.
